# Precursor‐Engineered Volatile Inks Enable Reliable Blade‐Coating of Cesium–Formamidinium Perovskites Toward Fully Printed Solar Modules

**DOI:** 10.1002/advs.202401783

**Published:** 2024-05-13

**Authors:** Tian Du, Viktor Rehm, Shudi Qiu, Subhajit Pal, Dongju Jang, Zijian Peng, Jiyun Zhang, Haozhen Yuan, Joe Briscoe, Wolfgang Heiss, Christoph J. Brabec, Hans‐Joachim Egelhaaf

**Affiliations:** ^1^ Forschungszentrum Jülich GmbH Helmholtz‐Institute Erlangen‐Nürnberg (HI ERN) Immerwahrstraße 2 91058 Erlangen Germany; ^2^ Department of Material Science, Materials for Electronics and Energy Technology (i‐MEET) Friedrich‐Alexander‐Universität Erlangen‐Nürnberg Martensstraße 7 91058 Erlangen Germany; ^3^ School of Engineering and Materials Science Queen Mary University of London London E1 4NS United Kingdom

**Keywords:** cesium formamidinium perovskite, reliable blade coating, single crystals, solar module, volatile ink

## Abstract

Reliable fabrication of large‐area perovskite films with antisolvent‐free printing techniques requires high‐volatility solvents, such as 2‐methoxyethanol (2ME), to formulate precursor inks. However, the fabrication of high‐quality cesium–formamidinium (Cs–FA) perovskites has been hampered using volatile solvents due to their poor coordination with the perovskite precursors. Here, this issue is resolved by re‐formulating a 2ME‐based Cs_0.05_FA_0.95_PbI_3_ ink using pre‐synthesized single crystals as the precursor instead of the conventional mixture of raw powders. The key to obtaining high‐quality Cs–FA films lies in the removal of colloidal particles from the ink and hence the suppression of colloid‐induced heterogeneous nucleation, which kinetically facilitates the growth of as‐formed crystals toward larger grains and improved film crystallinity. Employing the precursor‐engineered volatile ink in the vacuum‐free, fully printing processing of solar cells (with carbon electrode), a power conversion efficiency (PCE) of 19.3%, a *T_80_* (80% of initial PCE) of 1000 h in ISOS‐L‐2I (85 °C/1 Sun) aging test and a substantially reduced bill of materials are obtained. The reliable coating methodology ultimately enables the fabrication of carbon‐electrode mini solar modules with a stabilized PCE of 16.2% (average 15.6%) representing the record value among the fully printed counterparts and a key milestone toward meeting the objectives for a scalable photovoltaic technology.

## Graphical abstract

Upscaling the fabrication of solar modules is key to the commercialization of photovoltaic technology based on metal–halide perovskite materials. This study reports a reliable blade‐coating methodology for large‐area cesium–formamidinium (Cs–FA) perovskite films. This methodology enables a vacuum‐free, full printing of perovskite solar modules, which is directly transferrable to the roll‐to‐roll production line.

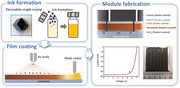

## 1 Introduction

Transferring from utilizing the spin‐coating technique to scalable printing techniques for the fabrication of metal–halide perovskite thin films represents a key milestone in realizing mass production of perovskite photovoltaics (PV). However, there is a noticeable gap in power conversion efficiencies (PCE) between the spin‐coated lab‐size solar cells and the printed solar modules, greater than that of other typical thin‐film PV technologies.^[^ ^1^ ^]^ The losses of PCE are arguably caused by a decline of film quality in the course of upscaled thin‐film fabrication, owing to a deficiency of reliable methods to control the crystallization of perovskites as the film area expands.^[^ ^2^, ^3^ ^]^ It has been essentially recognized that the emergence and the resolution of declined film quality are closely linked to the properties of the precursor inks.^[^ ^4^ ^]^ Therefore, the focus of this work is the re‐design of perovskite precursor inks that are both technically suitable and economically feasible for upscaling.

Rapid drying of the liquid film cast from precursor ink, which creates the necessary supersaturation, remains one of the pivotal physical processes in the crystallization of perovskite films. This is because sufficient supersaturation generates numerous amounts of perovskite nuclei, templating the formation of smooth, pinhole‐free, and highly crystalline solid films.^[^ ^5^ ^]^ In this context, owing to enhanced volatility, 2‐methoxyethanol (2ME) is favored over N,N‐dimethylformamide (DMF) as a matrix solvent (constituting typically 70–90% of the ink volume) for precursor ink formulation.^[^ ^6^, ^7^, ^8^, ^9^ ^]^ It is more so in the printing processing of perovskites where the well‐established “antisolvent quenching” method is not used and solvent extraction is alternatively achieved by continuous gas flow, known as “gas quenching.”^[^ ^10^, ^11^, ^12^ ^]^ On the other hand, rapid drying of the precursor ink accelerates coating speed and thereby increases the production capacity of perovskite film. Hence, the development of a volatile ink system is regarded as a roadmap toward reliable manufacturing of large‐area perovskite PV in the future.^[^ ^13^ ^]^ However, it is worth noting that perovskites fabricated with 2ME‐based inks thus far were mainly methylammonium lead iodide (MAPbI_3_)^[^ ^14^, ^15^, ^16^ ^]^ or methylammonium‐alloyed formamidine (MA‐FA) mixed perovskite^[^ ^17^, ^18^ ^]^ The fabrication of cesium–formamidine (Cs–FA) mix‐cation perovskites, which are thermally more stable than their MA counterparts and show optical band gaps closer to the optimal value of Shockley–Queisser limit,^[^ ^19^ ^]^ has been rarely reported.^[^ ^20^ ^]^ The deficiency in reports can be attributed to the inadequate solubility of cesium halides, such as cesium iodide (CsI), cesium bromide (CsBr), or cesium chloride (CsCl), in 2ME that hinders the formation of high‐quality, phase‐pure α‐FAPbI_3_ perovskite.^[^ ^21^ ^]^ More importantly, the lower solvating power of 2ME to perovskite precursors may cause unwanted changes in the solution chemistry that ultimately lead to deteriorated film quality, which is the main challenge to be addressed in this study.

Redissolving pre‐synthesized perovskite crystals in specific solvents emerged as an ink‐engineering strategy that yielded improvements in both film quality and the reproducibility of solar cell performance.^[^ ^22^, ^23^, ^24^, ^25^ ^]^ The technique was subsequently applied in the fabrication of state‐of‐the‐art perovskite solar cells.^[^ ^26^, ^27^ ^]^ In contrast to the conventional practice of mixing two or more raw powders, direct dissolution of perovskite crystals offers a straightforward method to prepare high‐purity inks with precise control over stoichiometry.^[^ ^28^ ^]^ Thin films prepared from this method usually exhibit improved optoelectronic qualities, primarily owing to the removal of impurity ions in the raw powders that would otherwise introduce electronic defects when they remain in the resulting film.^[^ ^29^, ^30^ ^]^ However, film processing in these works was based on spin‐coating and antisolvent‐quenching methods, from which the lessons learned may not be directly transferable to scalable printing methods of which the film formation mechanisms may be remarkably different. This can only be resolved by a comprehensive understanding of the underlying film‐formation mechanisms responsible for the reported film‐quality improvements. Unfortunately, questions remain regarding a couple of processes like how perovskite crystallization is altered by using high‐purity crystal precursors and how this alteration would then cause variations in grain size and film orientation. These aspects are clearly beyond the current understanding of simply removing the precursor impurities as electronic defects, which will be explored in this study.

Here, we address the above issues by demonstrating the blade‐coating of high‐quality Cs_0.05_FA_0.95_PbI_3_ films, utilizing 2ME‐based ink containing re‐dissolved Cs_0.05_FA_0.95_PbI_3_ single crystals as precursors. The use of single crystals does not improve the precursors’ solubility, but with the same precursor composition, it profoundly modifies the ink chemistry and thereby the crystallization kinetics of perovskites toward a remarkable improvement of film quality. We first show the reliability in achieving large‐area film homogeneity using 2ME‐based ink in gas‐quenching‐assisted blade coating, and then the improvement of solar cell performance by using single crystals as precursors, followed by an exploration of the variation in perovskite crystallization kinetics as the origin of improved thin‐film optoelectronic properties. We highlight the utilization of our precursor‐engineered volatile inks in the high‐throughput, vacuum‐free fabrication of carbon‐electrode perovskite mini modules, with a stabilized PCE of 16.2% achieved that is the highest value thus far among the fully printed counterparts. We conclude with a cost assessment of perovskite precursor materials derived from single crystal synthesis versus a direct mixture of raw powders, highlighting the economic feasibility of our precursor engineering method.

## 2 Results and Discussion

### 2.1 Impact of Matrix Solvent on Film Processing Window

Coating of large‐area perovskite films for optoelectronic devices requires control of both the morphological properties and microstructural properties. Morphologically the films need to have smooth surfaces, to be compact and free of pinholes. Microstructurally they should be highly crystalline, oriented, and defect‐less. In this work, film morphology over a large area is controlled by inducing high supersaturation uniformly in the as‐cast wet film through the use of volatile solvents. In the meanwhile, we optimize and fine‐tune the microstructure by extending the time window of crystal growth, achieved by employing redissolved single crystals as ink precursors.

**Figure 1:**
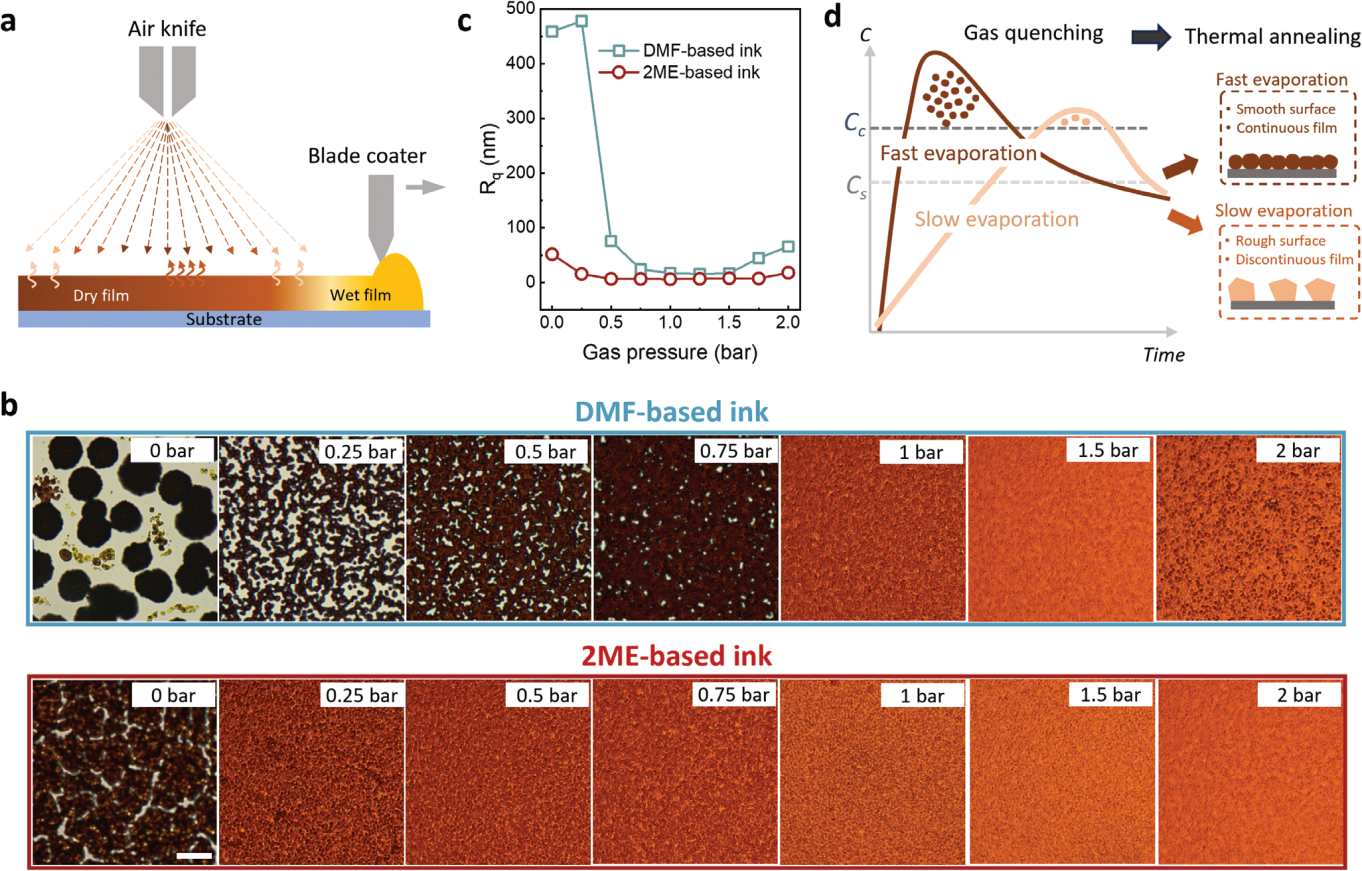
Origin of film inhomogeneity and comparison of ink processing window. a) Schematic drawing of the blade‐coating of perovskite film, the gas‐assisted liquid film drying (gas quenching) with an air knife, and the variation of local gas pressure on the film surface. b) Optical microscopic images, taken with light transmission mode, of the perovskite films fabricated with varied gas pressure. Each of the images covers a 50 × 50 µm area and the scale bar is 10 µm. The films are fabricated on 2.5 × 2.5 cm substrates with a pressure‐adjustable air gun directly above the substrates. c) Surface root‐mean‐square roughness (*R*_q_), measured with a confocal microscope from a 50 × 50 µm area of perovskite films fabricated from 2ME‐based ink and DMF‐based ink and dried with gas quenching treatment at varied gas pressure. d) LaMer diagram showing the crystallization kinetics of perovskites and the sketch of final film morphologies in case of fast and slow solvent evaporation. *C_c_* is critical concentration and *C_s_* is saturation concentration.

We start by considering the impact of ink volatility on the macroscopic homogeneity in typical printing of large‐area perovskite films. The precursor inks are formulated with the volatile 2‐methoxyethanol (2ME) as matrix solvent, as compared with the conventional and less‐volatile N,N‐dimethylformamide (DMF). Both 2ME and DMF are blended with a small quantity of N‐methyl‐2‐pyrrolidone (NMP) as the coordinating solvent, they are thereafter referred to as 2ME‐based ink and DMF‐based ink, respectively. We depict in **Figure** **1**a the process of blade coating of perovskite precursor ink on the substrate and the subsequent drying of the liquid film by gas quenching. Gas quenching treatment of the liquid film prior to thermal annealing is a widely adopted technique in perovskite film printing to ensure a uniform film with full coverage on the substrate,^[^ ^31^ ^]^ as is like the well‐known “antisolvent quenching” in spin coating. In this process, the pressure of the applied gas, which dedicates the rate of solvent extraction, is arguably the most critical factor determining the final film's morphology.

In Figure 1b, we visualize the variations in macroscopic film morphology as a function of varying gas pressure through a series of optical microscopic images. We quantify these morphological changes in Figure 1c by measuring the surface root‐mean‐square roughness (*R*q) using a confocal microscope (shown in Figure S1, Supporting Information). For DMF‐based ink, lowering the gas pressure leads to the formation of a discontinuous film comprising large but isolated grains (appearing as dark regions in optical microscopic images) with surrounding pinholes (appearing as white regions). By increasing gas pressure, compact films free of pinholes are formed and their surface roughness is reduced. As the gas pressure is further increased, film roughness increases again, and this is because the strong gas flow can induce hydrodynamic perturbations on the liquid film surface. Comparatively, the films derived from 2ME‐based ink show much less morphological variation as the gas pressure is decreased, and a lower minimum gas pressure (i.e. 0.25 bar) is needed to ensure film compactness. This implies that, in terms of assuring film compactness, the volatile 2ME‐based ink offers a wider processing window against any variations of local gas pressure than DMF‐based ink. Given that the as‐cast wet film needs to be timely treated by gas quenching to prevent unwanted natural drying, which can be realized by attaching an air knife with the coating bar in sheet‐to‐sheet coating or through a fixed air knife right behind the slot‐die coater in roll‐to‐roll production, the volatile 2ME‐based ink can overcome local variation of gas pressure during gas‐quenching‐assisted large‐area film coating process resulting in an improvement of macroscopic film homogeneity.

The increased volatility of 2ME originates from its higher vapor pressure (0.82 vs 0.35 Pa at 20 °C) and weaker coordinating power to Pb^2+^ than DMF, making it easier to escape from the wet film.^[^ ^32^, ^33^ ^]^ The reduction of ink volatility that is more likely to destroy thin‐film compactness and smoothness is illustrated in Figure 1d using a LaMer graph and a schematic drawing:^[^ ^34^ ^]^ Rapid solvent extraction can increase wet film concentration (*C*) drastically above the critical concentration (*C_C_*) within a short period of time, leading to a high supersaturation in the liquid film and, consequently, a high density of perovskite nuclei.^[^ ^5^ ^]^ Slow solvent evaporation cannot create high‐level supersaturation due to the simultaneous consumption of solutes, resulting in a substantially lower density of perovskite nuclei. When transferring these gas‐quenched films to subsequent thermal annealing, their crystallizations are then dictated by diffusion‐controlled growth of the existing crystals, and the formation of additional nuclei is largely inhibited. The high concentration of nuclei, and therefore a dense crowd of perovskite nanocrystals formed from the liquid phase, templates the growth of a compact polycrystalline film fully covering the substrate. In contrast, the sparsely distributed nuclei generated during slow evaporation evolve into large but isolated grains, resulting eventually in discontinuous films with high surface roughness after thermal annealing.

### 2.2 Impact of Precursor Source on Solar Cell Performance

**Figure 2:**
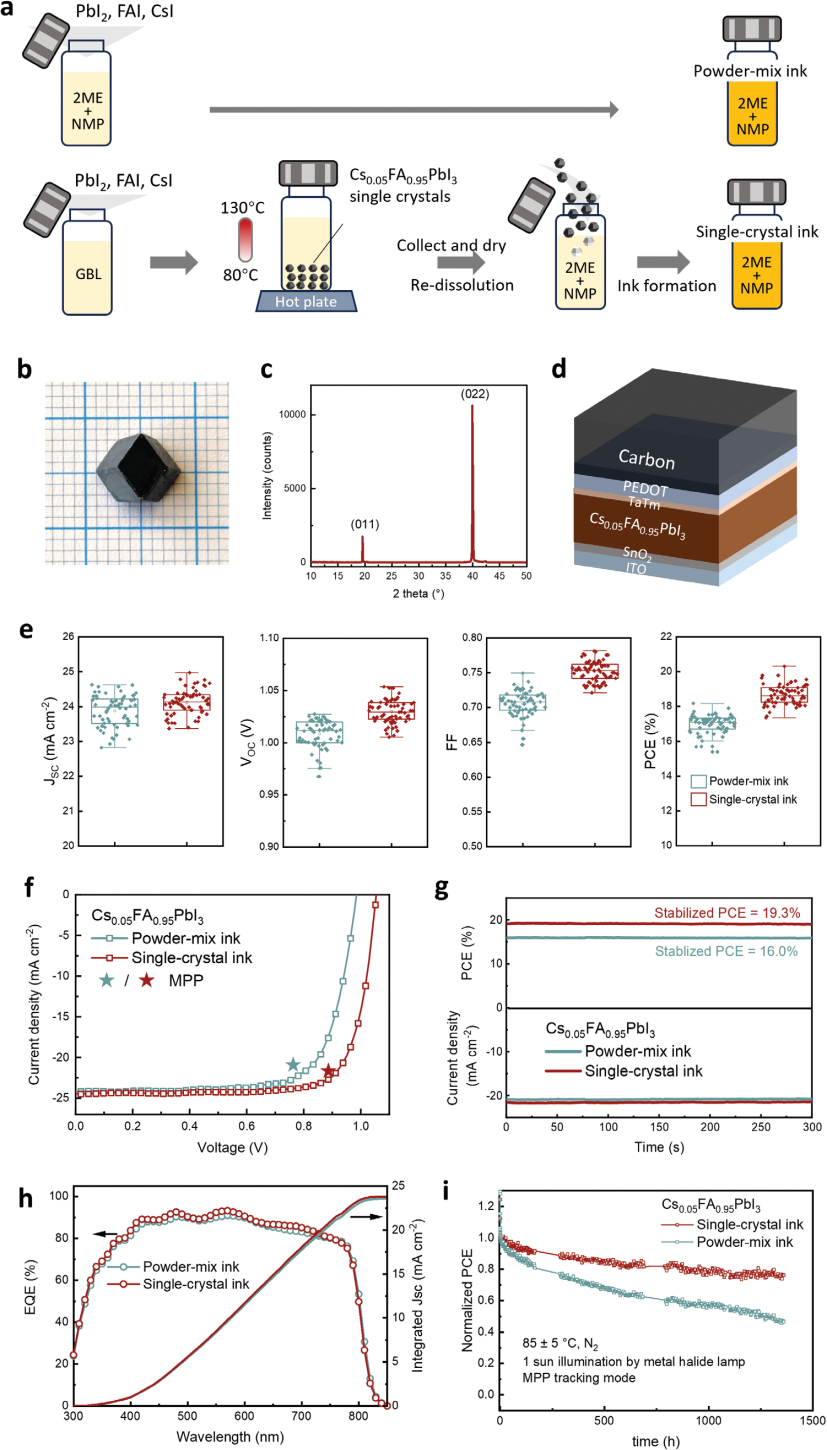
Single crystals synthesis, ink formation, and solar cell performance. a) Schematic illustration of the synthesis of Cs_0.05_FA_0.95_PbI_3_ single crystals and the redissolution of the obtained single crystals in mixed solvents of 2ME and NMP (9:1 in volume ratio) to form precursor ink, as compared to the conventional mix of PbI_2_, FAI, and CsI in the same solvent to form precursor ink. b) Photograph of a representative Cs_0.05_FA_0.95_PbI_3_ single crystal. c) XRD pattern of the {011} family of planes of the Cs_0.05_FA_0.95_PbI_3_ single crystal. d) Schematic illustration of the layer configuration of the carbon‐electrode perovskite solar cells. e) Statistical data of *J*_SC_, *V*_OC_, FF, and PCE of the PSC with perovskite films derived from single‐crystal ink and powder‐mixing ink. The PV parameters are extracted from reverse‐scan (i.e., from open circuit to short circuit) JV measurement, and the data are collected from 60 devices for each condition. f) JV curves of the champion solar cells, both measured with reverse scan with a speed of 100 mV s^−1^. The star indicates the MPP of each cell. g) Time‐dependent photocurrent and corresponding PCE of the champion cells held at the voltage of MPP. h) EQE spectra and integrated J_SC_s of the two champion solar cells. i) Evolution of PCE of the solar cells with perovskite films fabricated from single‐crystal ink and powder‐mix ink. The aging test is carried out in a nitrogen‐filled box at 85 ± 5 °C under 1 sun equivalent illumination, provided by a metal halide lamp with a UV filter. The PCE of each device is normalized to the respective value as the temperature reaches 85 °C.

Having shown the importance of ink volatility and the suitability of 2ME in upscaled fabrication, we turn to the fabrication of Cs–FA perovskites with only 2ME‐based ink, comprising a blend of 2ME and NMP with a volume ratio of 9:1, a value both adopted in literature and optimized in‐house (Figure S2, Supporting Information).^[^ ^7^, ^35^ ^]^ When mixing the raw powders, namely lead iodide (PbI_2_), formamidinium iodide (FAI), and cesium iodide (CsI), we observed precipitation upon adding more than 5%_mol_ of CsI, Figure S3 (Supporting Information), indicating the limited solubility of CsI in 2ME‐based ink. We note that increasing the volume ratio of NMP over 1:9 may slightly increase the amount of dissolved CsI, but it leads to rapid coarsening of the film surface as we found during our in‐house optimization of film coating, whilst higher NMP volume ratio also remarkably reduces ink volatility. We therefore focus on Cs_0.05_FA_0.95_PbI_3_ as a benchmark composition for the reference ink. We formulate the target ink by redissolving Cs_0.05_FA_0.95_PbI_3_ single crystals, which have been pre‐synthesized with a so‐called “inverse temperature crystallization (ITC)” method, as we illustrate in **Figure** **2**a. In brief, the synthesis involves co‐dissolving PbI_2_, FAI, and CsI in γ‐butyrolactone (GBL), followed by a gradual temperature increase from 80 to 130 °C during which black perovskite crystals nucleate and grow from the solution, depicted in Figure S4 (Supporting Information). The yield of crystals is 37% and the obtained crystals typically have a dodecahedral shape, as seen in Figure 2b. X‐ray diffraction measurement of the {011} planes of the crystal, plotted in Figure 2c, shows only (011) and (022) diffraction peaks with no other peaks discernible, indicating the single crystalline nature of the obtained crystal.

We then evaluate the performance of solar cells comprising perovskite films prepared from the powder‐mix (PM) ink and the single‐crystal (SC) ink. Both sets of films are treated by gas quenching with a gas pressure of 1 bar. We depict in Figure 2d the layer configuration of our prototype solar cell,^[^ ^36^ ^]^ and it is worth noting that all layers, including the counter carbon electrode, are processed with printing technique in ambient conditions with controlled (i.e. < 5% RH) humidity. We plot in Figure 2e the statistical data of the PV parameters, namely the short‐circuit current density (J_SC_), open‐circuit voltage (V_OC_), fill factor (FF), and power conversion efficiency (PCE), which clearly show that the solar cells fabricated with SC ink outperform those from PM ink. The improvement in performance is manifested by an increase in both V_OC_ and FF, resulting in an average increase of PCE from 16.7% to 18.7%.

The current–voltage (JV) measurement of the champion cells, Figure 2f, shows an increase of PCE from 17.4% (PM ink) to 20.1% (SC ink). Since hysteresis in the JV curves is observed in both devices, Figure S5 (Supporting Information), we perform quasi‐steady‐state JV scans at a speed of 1 mV s^−1^, which allows us to determine their maximum power points (MPP). We then measure the photocurrent and the corresponding stabilized PCE by holding the solar cell at the voltage of MPP. In Figure 2g, we observe that the stabilized PCE of the champion cells prepared from PM and SC inks are 16.0% and 19.3%, respectively. Although the stabilized PCEs are slightly lower than the respective values measured from the JV scan, it underlines a robust improvement of solar cell performance using precursor ink that contains redissolved single crystals. The external quantum efficiency (EQE) spectra of the two champion devices, Figure 2h, show that the integrated J_SC_s are 23.6 mA cm^−2^ for PM ink and 23.8 mA cm^−2^ for SC ink, respectively. These values reasonably agree with the J_SC_s measured from the JV scans (24.1 and 24.4 mA cm^−2^).

The operational stability of the unpackaged solar cells in the N_2_ atmosphere is characterized by showing the evolution of normalized PCE in Figure 2i, the original data, and other photovoltaic parameters in Figure S6 (Supporting Information). The solar cells both undergo aging at 85 °C with 1 sun equivalent illumination using maximum power point tracking mode, aligned with the so‐called “ISOS‐L‐2I” testing protocol.^[^ ^37^ ^]^ A drop of PCEs in the initial 30 min is observed in both cells due to temperature escalation, Figure S7 (Supporting Information). Nevertheless, the perovskite films derived from SC ink exhibit a noticeable improvement in stability with ≈80% of initial PCE (*T_80_*) maintained after 1000 h, as compared to the *T_80_* of approximately only 200 h for the perovskite derived from PM ink. Moreover, a consistent improvement of film stability in ambient conditions is observed, shown in Figure S8 (Supporting Information). These findings highlight that engineering the precursor ink by dissolution of pre‐synthesized single crystals, instead of the conventional mixture of raw powders, not only robustly enhances solar cell performance but also improves the solar cell's operational stability.

### 2.3 Impact of Precursor Source on Film Crystallization Kinetics

**Figure 3:**
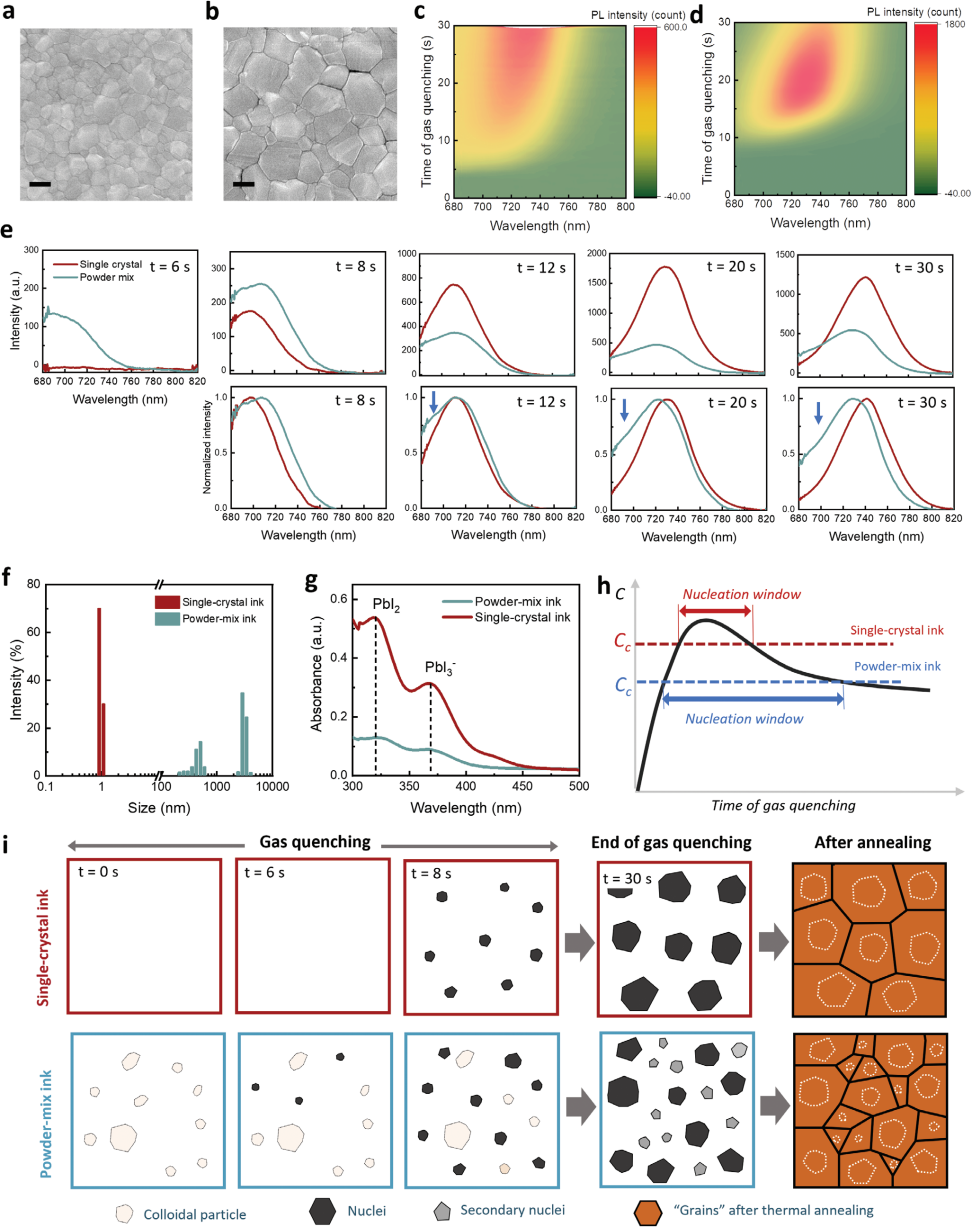
Impact of precursor source on solution chemistry and crystallization kinetics. a, b) Surface SEM images of perovskite films derived from powder‐mix ink (a) and single‐crystal ink (b). The scale bars on both images are 100 nm. c, d) Contour maps of the evolution of PL spectra during the 30‐s gas quenching treatment of liquid films cast from PM ink (c) and SC ink (d). The samples are excited by a 532‐nm laser diode. e) Selected plots of PL spectra (top panel) and normalized PL (bottom panel) spectra at the 6th, 8th, 12th, 20th, and 30th second of the gas quenching treatment. The arrow indicates the broadening of the PL peaks. f) Colloidal hydrodynamic size distribution of the perovskite inks. g) Absorption spectra of the perovskite inks. h) Schematic drawing of LaMer diagrams showing the evolution of concentration with time during the drying process for liquid films cast from SC ink and PM ink. i) Schematic drawing of the nucleation and growth of perovskite crystals during gas quenching, and the morphology of films after thermal annealing.

We observe a clear morphological change between the film derived from powder‐mix ink (PM film) and the film derived from single‐crystal ink (SC film) from surface scanning electron microscopy (SEM) images, **Figure** **3**a,b, which shows an increase of average grain size from 90 ± 47 to 250 ± 74 nm (see also Figure S9, Supporting Information). The increase in lateral grain size is accompanied by the emergence of monolithic grains in the out‐of‐plane direction in the SC film (Figure S10, Supporting Information). We move on to unravel how the choice of precursor source influences the crystallization kinetics of perovskites which leads to changes in grain size. As mentioned earlier, gas quenching is arguably the most important process in determining the properties of the resulting films, this process is therefore scrutinized through an in situ photoluminescence (PL) measurement that we developed previously.^[^ ^38^ ^]^ Key observations from the in situ measurement include the emergence of PL peaks that signifies the formation of perovskite nanocrystals as nuclei, whereas any shift of the peak positions toward longer wavelengths is ascribed to a size effect that correlates to the growth of the nanocrystals.^[^ ^39^ ^]^ In Figure 3c,d, we show the contour plots of spectral evolution during the 30 s gas quenching treatment. The key difference observed is an early rise of PL, a wider spectral distribution of the emission peak, and an overall lower intensity for the PM film as compared to the SC film.

To probe in detail the variation in crystallization kinetics, we display in Figure 3e the original PL spectra along with the normalized spectra at selected time points from Figure 3c,d. At the sixth second since turning on the gas flow, a PL peak emerges from the PM film whereas no signal is seen from the SC film until the eighth second. However, starting from the eighth second, the intensity of the PL peaks measured from the SC film rises drastically, and their intensities are ≈2–3 times higher than those measured from the PM film by the end of the gas‐quenching. An analysis of the normalized spectra of the SC film shows that all the peaks closely approximate a Gaussian shape and shift toward longer wavelengths at a constant speed. In strong contrast, the PL peaks of the PM film are asymmetric at the beginning and become more so toward the end of gas quenching, showing an abnormal broadening with a “shoulder” emerging on the short‐wavelength side.

The results suggest a clear change in perovskite crystallization kinetics depending on the precursor source: The delayed appearance of the PL peak suggests a delay in the initiation of nucleation at higher supersaturation levels, indicating a higher critical concentration for nucleation (*C_c_*) in the SC liquid film; the constant pace of peak red‐shifting indicates uniform growth of all formed nanocrystal; the emergence of peak broadening at the short‐wavelength side is attributed to secondary nucleation, resulting in additional formation of nanocrystals in parallel to the growth of existing crystals. The strong peak asymmetry at the end of gas quenching correlates therefore to a broader distribution of crystal sizes, particularly an abundance of small crystals in the dried film. The drop of PL intensity in both films at a later stage of the gas quenching is most likely caused by defect formation at crystal boundaries when the nanocrystals meet each other.^[^ ^40^ ^]^ The relatively higher PL intensity, however, indicates a smaller trap density in the crystals formed in SC ink.

To understand the underlying chemical factors responsible for the above‐described variations in crystallization kinetics, we investigate the formation of colloidal particles dispersed in the precursor ink using dynamic light scattering (DLS). We plot in Figure 3f the DLS intensity distribution, revealing the presence of large colloidal particles in the PM ink with their hydrodynamic sizes ranging from a few hundred nanometres to a few micrometers, contrary to the small colloidal particles of a few nanometres with a remarkably narrowed size distribution observed in the SC ink. Furthermore, the two precursor inks also show a clear difference in the absorption spectra, Figure 3g, manifested by an enhancement of the absorption peaks at 325 and 375 nm in the SC ink. The 375 nm peak is assigned to a soluble PbI_3_^−^ plumbate formed by the complexation between PbI_2_ and I^−^.^[^ ^41^ ^]^ The quenching of the 375 nm peak, typically seen in a PbI_2_‐rich state of a solution,^[^ ^41^, ^42^ ^]^ suggests that these colloidal particles are essentially the local aggregates of PbI_2_ decorated by organic ions.^[^ ^43^ ^]^ We propose that the large particles dispersed in the PM precursor ink emerge as heterogeneous nucleation centers, effectively reducing the surface energy of the nucleus and thereby also reducing the energy barrier of perovskite nucleation for the latter.^[^ ^44^ ^]^

We can then summarize the variation in crystallization kinetics within the framework of the LaMer mechanism, as illustrated in Figure 3h: At the beginning of gas quenching, the strong air efficiently removes 2ME from the wet films, allowing both films to reach high concentrations within a short period of time. With most 2ME removed and the emergence of perovskite nuclei, the concentration of solutes reaches a maximum before starting to decrease. Nucleation occurs until *C* drops below *C_c_*, a threshold concentration for the nucleation of a new phase, film crystallization is thereafter dominated by diffusion‐controlled growth of the existing crystals. The key parameter that is varied here is the *C_c_*, which is higher for SC ink than for PM ink as highlighted in Figure 3h, due to a reduced energy barrier for nucleation for the latter. A higher *C_c_* means delayed onset of perovskite nucleation and a shorter time window for nucleation. In contrast, a lower *C_c_* results in a longer time window for nucleation, during which secondary nucleation competes with the growth of the existing nanocrystals.

The variation in microstructural properties can be therefore illustrated in Figure 3i: In the case of SC ink, shortly after the initial nucleation, additional nucleation is otherwise suppressed and the crystallization is thereafter dominated by the diffusion‐controlled growth of the as‐nucleated nanocrystals until the end of gas quenching. Thermal annealing completely removes the residual solvent (which is mainly NMP), converting the remaining non‐perovskite phase, as indicated by the XRD diffraction pattern (Figure S11, Supporting Information), and amorphous phase in the gas‐quenched film into perovskite. This leads to further crystal ripening and their binding into “grains.” Importantly, the extended time window for crystal growth leads to not only an increase of crystal/grain size in the resulting film but also the healing of local defective clusters leading to an improvement of film crystallinity.^[^ ^45^ ^]^ In contrast, the wet film cast from PM ink undergoes a prolonged nucleation time window. The continuous formation of new crystals (secondary nuclei) leads to an extraordinarily high density of nuclei. Consequently, the individual nuclei can only grow up to a certain extent until they meet each other, with their growth impeded by the formation of new nuclei. Despite forming a compact film, the lack of ripening of these crystals results in smaller crystal/grain sizes in the resulting film as well as inferior film crystallinity.

The emergence of large colloidal particles in precursor solution as unwanted heterogeneous nucleation sites has been previously shown.^[^ ^46^, ^47^ ^]^ It is also reported that the dissolution of these colloidal particles fosters increased crystal/grain size, improved film crystallinity, and improvement of optoelectronic properties of the perovskite films, which agrees with our observation. In our case, the emergence of unwanted particle dispersion can be attributed to trace impurities in the raw powders, namely the insoluble Pb(H_2_PO_3_)_2_^[^ ^48^ ^]^ and Pb(OH)I found in even high‐purity PbI_2_,^[^ ^49^ ^]^ that can induce non‐negligible aggregation of PbI_2_. In the meanwhile, the limited coordination capability of 2ME to Pb^2+^ can be a major cause of the high sensitivity of particle formation to these insoluble impurities,^[^ ^33^ ^]^ and the necessity of employing single crystals as precursors in order to form a clean, particle‐free ink. Such a hypothesis can be supported by a comparative investigation of the DMF‐based inks: With stronger coordination with Pb^2+^ that can break up the PbI_2_ aggregates, we found that the DMF‐based powder‐mix precursors show much smaller colloidal size of a few nanometres, with no noticeable difference to that using single‐crystal precursors, depicted in Figure S12 (Supporting Information). They also show nearly identical absorption spectra featuring a strong PbI_3_^−^ absorption peak, displayed in Figure S13 (Supporting Information). These findings are consistent with a clear suppression of heterogeneous nucleation when employing the same powder‐mix precursors in a DMF‐based ink, shown in Figure S14 (Supporting Information). Furthermore, there is a smaller, although still noticeable, discrepancy in the performance of solar cells prepared from DMF‐based inks containing powder mix or single crystals, the plotted in Figure S15 (Supporting Information).

These findings imply that the insoluble impurities in the high‐purity PbI_2_ (namely, Pb(H_2_PO_3_)_2_, Pb(OH), etc) do not directly form point defect sites, as contrary to the impurity ions (such as K^+^, Ag^+^) in low‐purity PbI_2_,^[^ ^50^ ^]^ but they remarkably alter the solution chemistry by inducing the formation large colloidal particles. Our results also highlight a trade‐off in perovskite precursor ink formulation: The less volatile, Lewis base DMF that shows stronger coordinating power with Pb^2+^ can benefit laboratory cell fabrication but may not be desirable for upscaling; Using volatile 2ME with a lower boiling point and a lower Pb^2+^ coordination is more suitable for achieving macroscopic film homogeneity in upscaled fabrication, but it may introduce increased complexity in solution chemistry that can remarkably affect the film's microstructural properties. Consequently, the engineering of precursors plays a more significant role in obtaining high‐quality perovskite films for the precursor ink with volatile solvents.

Concerning the reports of efficient solar cells fabricated with 2ME‐based ink with powder‐mix precursors, primarily involving MAPbI_3_ or FA‐MA mix perovskites, it is worth an extended discussion that the deficiency in crystal growth observed in our PM ink is inherently caused by a deficiency of Cs alloying to FAPbI_3_ perovskite.^[^ ^21^, ^51^ ^]^ That is to say, if a greater amount of Cs (i.e. > 5%_mol_) can be included in the precursor ink, we shall substantially improve the crystal growth rate that surpasses the rate of heterogeneous nucleation, leading to improved film crystallinity. To test this hypothesis, we measure the films with reduced Cs alloying, finding that the Cs_0_FAPbI_3_ film shows no detectable PL throughout the course of gas quenching whilst the Cs_0.02_FA_0.98_PbI_3_ film shows PL peaks with rather low intensity and negligible redshift, Figure S16 (Supporting Information). On the contrary, by alloying MA to FAPbI_3_ to a level 10 and 25%_mol_ in the 2ME‐based PM ink, we observe a noticeable enhancement of crystal growth over secondary nucleation, manifested by a stronger PL intensity, a greater magnitude of redshift and a disappearance of peak broadening, as shown in Figure S17 (Supporting Information). These results highlight the critical role of the appropriate level of small cation alloying, by either Cs or MA, in driving the crystal growth of FAPbI_3_ and thereby in achieving high‐crystalline, photoactive α‐FAPbI_3_ perovskite.^[^ ^21^ ^]^ In the case of Cs_0.05_FA_0.95_PbI_3_, Cs alloying is constrained by its solubility leading to inherent limitation of crystal growth. Film quality thus becomes exceedingly sensitive to the emergence of heterogeneous nucleation that could otherwise prohibit indispensable crystal growth. Therefore, in the context of Cs_0.05_FA_0.95_PbI_3_ perovskite, the engineering of precursor to reactivate crystal growth by suppressing heterogeneous nucleation is a factor of utmost importance for obtaining high‐quality films.

### 2.4 Characterization of Film Properties

**Figure 4:**
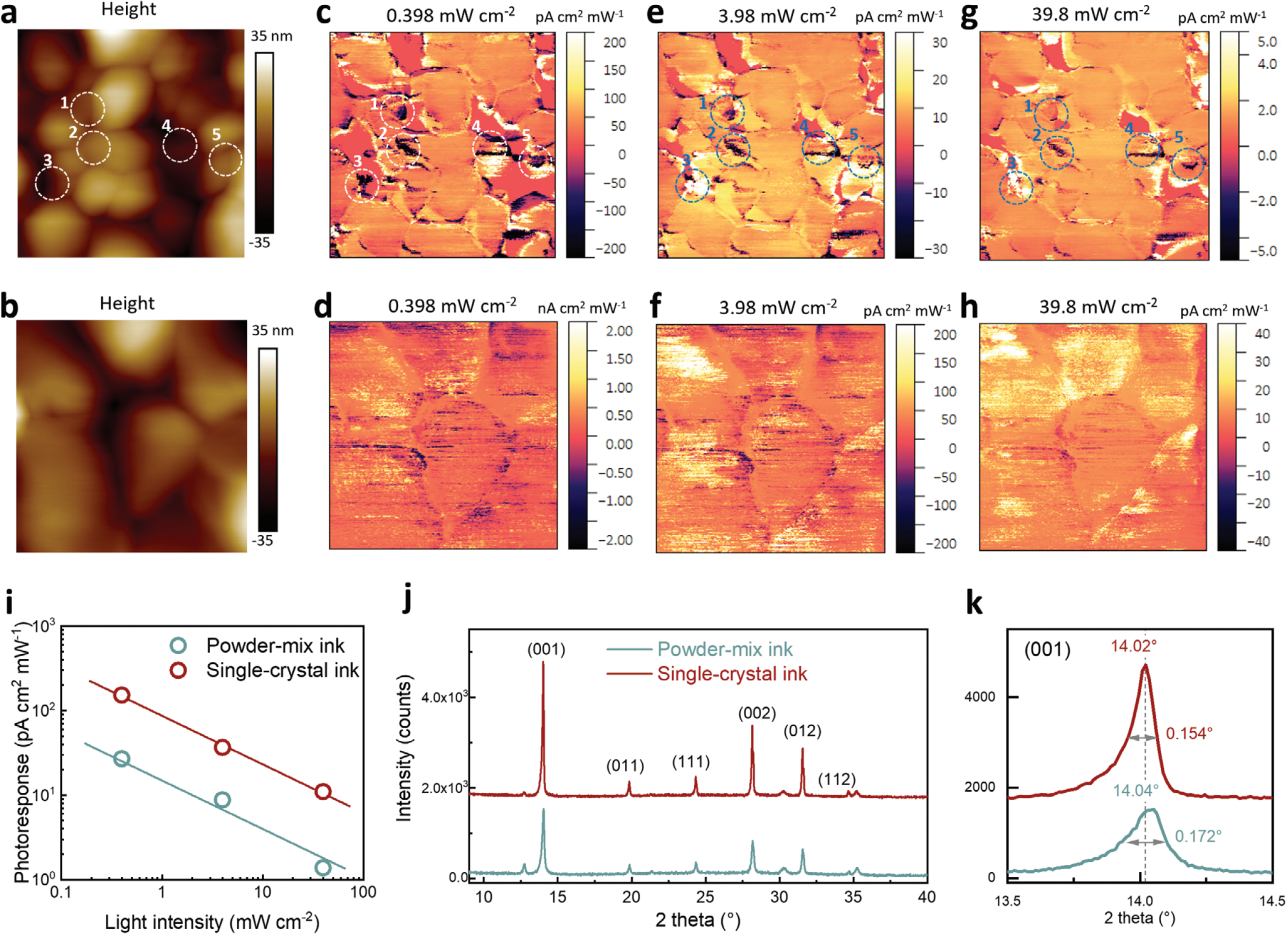
Characterization of film properties and local defective clusters. a,b) Surface height maps of perovskite films derived from powder‐mix ink (a) and single‐crystal ink (b). c–h) Photoresponse maps of the PM perovskite film (c, e, g) and the SC perovskite film (d, f, h) at varied illumination intensities of 0.398 (c, d), 3.98 (e, f) and 39.8 mW cm^−2^ (g, h). The circulated areas in Figure a, c, e and g, labeled “1–5,” indicate local defective clusters in the PM perovskite film. The illumination is provided by a light‐emitting didoe (LED) with a wavelength of 529 nm, and the intensity of incident light is tuned with neutral density filters. The bias applied on the pc‐AFM tip is 1.5 V. i) Area averaged photoresponse extracted from the mapping measurements. j) X‐ray diffraction patterns of perovskite films derived from powder‐mix ink and single‐crystal ink. k) Zoom‐in image of the (001) diffraction peak.

We now focus our investigation on the formation of local defective clusters and variation in film crystallinity due to a deficiency in crystal growth. The local optoelectronic properties of these films are characterized using photoconductive atomic force microscopy (pc‐AFM). In this method, the current passing through the films in the dark (*J_dark_*) and under illumination (*J_ph_*) of varied levels of incident light (*P_in_*) is measured, as shown in Figure S18 (Supporting Information). The metal tip/perovskite/ITO configuration essentially forms a photodetector, in which we determine the photoresponse [nA cm^2^ mW^−1^] of the perovskite films. The parameter is defined as (*J_ph_* − *J_dark_*) / *P_in_*, quantifying the photocurrent generated per unit power of incident light, which is an assay of the films’ local photon‐to‐electron conversion properties.^[^ ^52^ ^]^ We display in **Figure** **4**a,b the surface height maps of a PM perovskite film and an SC perovskite film, and in Figure 4c–h the corresponding photoresponse maps under illumination of 0.389, 3.89, and 38.9 mW cm^−2^.

The local variation of optoelectronic qualities can be clearly visualized from the photoresponse maps. Under 0.398 mW cm^−2^ illumination, the photoresponse of the PM film (Figure 4c) shows a selection of local regions that exhibit negligible or even negative photoresponse, indicated by white circles on the image. These regions are essentially the local areas of low photoactivity. As illumination intensity increases to 3.98 mW cm^−2^ (Figure 4e) and further 39.8 mW cm^−2^ (Figure 4g), these local regions appear brighter as compared to their surrounding areas indicating an improvement of photoactivity. It suggests that the performance‐limiting factor primarily affects film under weak illumination levels and thus is likely related to trap‐mediated charge recombination.^[^ ^53^ ^]^ A detailed comparison between the height map (Figure 4a) and the photoresponse maps (Figure 4c,e,g) shows that these defective clusters are typically aligned with areas of concentrated small grains or at the boundaries of neighboring grains. In contrast, the defective clusters typically observed in PM films are less pronounced in the SC film, resulting in the significantly improved spatial uniformity of the photoresponse (Figure 4d,f,h). In Figure 4i, we plot the area‐averaged photoresponse data from the mapping measurements. While the photoresponse decreases similarly in both films as illumination levels are elevated, which is owing to an increase in bimolecular recombination,^[^ ^54^ ^]^ the SC film displays nearly a tenfold improvement in photoresponse, strongly indicative of its superior optoelectronic quality compared to the PM film.

X‐ray diffraction patterns, Figure 4j, reveal an approximately twofold higher diffraction intensity for all the diffraction peaks in SC film compared to PM film. A closer inspection of the (100) diffraction peak, Figure 4k, shows a noticeable reduction of peak FWHM (full width at half maximum) by 0.02° and a shift of peak position by 0.02° toward lower 2θ values. In case both films are of comparable thickness (≈750 nm), the increase of diffraction intensity is indicative of improvement of film crystallinity. The reduction of peak FWHM indicates an increase in crystal size in the out‐of‐plane direction with respect to the substrate. The peak shift toward lower 2θ correlates to an increase in out‐of‐plane lattice spacing. In the case of fixed perovskite composition, it indicates reduced compressive strain in the out‐of‐plane direction that is possibly caused by a reduction of residual in‐plane tensile strain realized by extended crystal growth.^[^ ^51^ ^]^ The XRD results qualitatively align with the change of grain size shown in Figure 3a,b, and are consistent with our proposed model of nucleation and crystal growth depicted in Figure 3i.

We then conclude that whilst the gas‐quenching‐induced primary nucleation templates the growth of pinhole‐free film, the subsequent continuous secondary nucleation may adversely impede the growth of the as‐formed crystals, resulting in inferior film crystallinity and smaller grains after thermal annealing. Importantly, the areas surrounding fragmented grains are identified as defective clusters limiting the optoelectronic properties of the perovskite film, showing that the origin of degraded film quality is a deficiency in local crystal growth. In this regard, extending crystal growth by suppressing the secondary nucleation premise is an effective defect‐management approach that promotes the formation of larger grains and the elimination of local defective clusters.

### 2.5 High Throughput Module Fabrication from Single‐Crystal Ink

**Figure 5:**
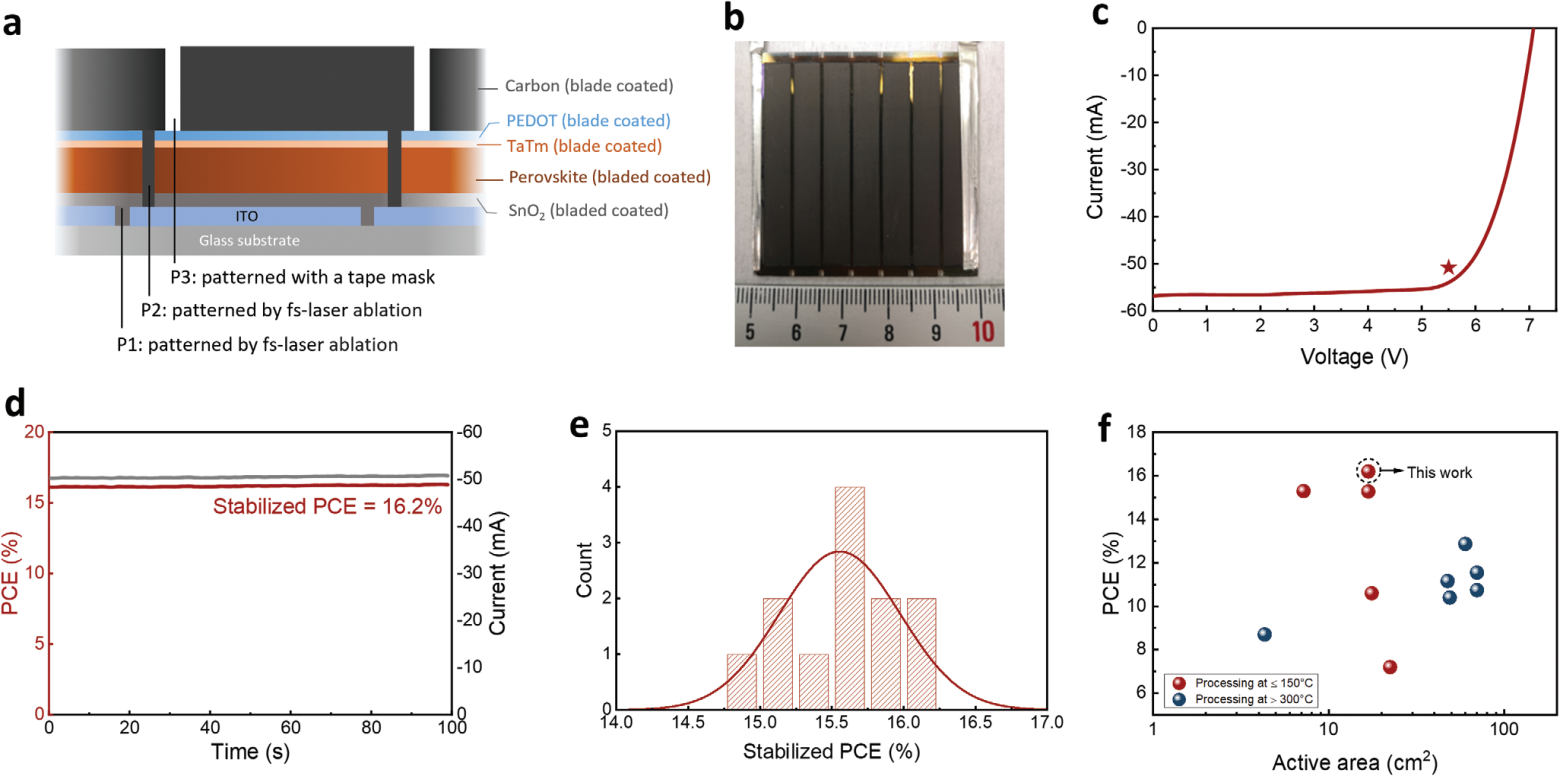
High‐throughput fabrication with single‐crystal ink. a) Schematic illustration of the interconnections of the fully printed perovskite mini solar module. b) Photograph of the perovskite mini solar module. c) JV curve (measured in reverse‐scan direction) and MPP of the mini solar module of champion PCE. d) Photocurrent and the stabilized PCE of the champion module were measured at MPP. e) Statistical data of the stabilized PCE measured from 12 mini solar modules. f) Summary of PCE versus active area of vacuum‐free, fully printed solar modules with carbon counter electrodes.

Finally, we demonstrate the high‐throughput production of solar modules utilizing single‐crystal ink. The layer configuration of the solar module is identical to that of the solar cell as illustrated in Figure 2d. The schematic diagram of the interconnection area of the solar module, **Figure** **5**a, shows the positions of the P1, P2, and P3 lines, which are needed for serially connecting two adjacent cells. The mini‐module is fabricated on a 5 × 5 cm^2^ substrate consisting of seven sub‐cells, Figure 5b, with an aperture area of 20.25 cm^2^, an active area of 16.84 cm^2,^ and a geometric fill factor of 83.2%. We plot in Figure 5c the JV scan and the MPP of the best‐performing mini solar module, which shows an active‐area PCE of 17.7% as derived from the JV curve. The stabilized PCE, measured with the same protocol as with the solar cells, shows an active‐area PCE of 16.2%, Figure 5d, Measurement of 12 modules, Figure 5e and Table S1 (Supporting Information), shows an average stabilized PCE of 15.6 ± 0.4%, showing satisfyingly high reproducibility of 2ME‐based single‐crystal ink in module fabrication. The reproducibility is arguably ascribed to the excellent processing window of 2ME‐based ink. In Figure 5f and Table S2 (Supporting Information), we summarize the PCE versus active areas of perovskite solar modules fully fabricated with vacuum‐free, printing techniques. The result shows that, to the best of our knowledge, we have by far presented the highest PCE reported for fully printed perovskite mini solar modules.^[^ ^55^ ^]^ The small PCE loss, 19.3% to 16.2%, during device upscaling from 0.06 to 16.84 cm^2^ underscores the reliability of our proposed volatile ink strategy in upscaling, whereas the processing at no greater than 150 °C makes it compatible with roll‐to‐roll production. The realization of full printing for efficient mini solar modules represents a critical milestone toward the gate for industrial commercialization of single‐junction perovskite cells.

In parallel to an improvement of solar cell PCE and operational stability, the utilization of Cs_0.05_FA_0.95_PbI_3_ single crystals enables a ≈80% reduction of the cost of perovskite precursor materials compared to using raw powders, from 23.3 to 4.5 Euros per gram based on the calculation of catalog prices of these materials from the manufacturers and a yield of 37% in single crystal synthesis, Tables S3 and S4 (Supporting Information). This cost advantage arises from the utilization of low‐purity precursor powders in the synthesis of single crystals, which are substantially more affordable than the high‐purity ones required for film fabrication with powder‐mixing ink. Although the cost of perovskite precursor may not be the major component in the total fabrication cost of fully printed modules,^[^ ^56^ ^]^ it is still worth highlighting that the synthesis of single crystals represents a more cost‐effective method for purifying perovskite precursors than the conventional purification processes involving individual raw powders. The production of high‐purity PbI_2_, FAI, and CsI is much more expensive as it involves energy‐intensive purification methods such as vacuum sublimation. Rather, during single crystal synthesis, all PbI_2_, FAI, and CsI can be facilely separated from the impurities at the same time. This is because only the perovskites show retrograde solubility in GBL, meaning that their solubility decreases with the increase in temperature. On the contrary, most of the common impurities in raw powder precursors (such as lead acetate, sodium acetate, potassium acetate, and potassium iodide^[^ ^50^ ^]^) have a higher solubility at elevated temperature, as opposite to the perovskites. Consequently, these impurities remain in the GBL solution, while perovskite single crystals precipitate. It is also worth noting that the synthesis of a single crystal itself is facile and, in principle, can be upscaled. Hence, the improvement in solar cell PCE and stability, coupled with the reduction in materials cost, underscores the cost‐effectiveness of employing single‐crystal inks for the upscaling of perovskite PV.

## 3 Conclusion

We first demonstrated the superior fabrication reliability of 2ME‐based precursor ink over the conventional DMF‐based precursor ink in gas‐quenching‐assisted blade coating of perovskite films. We then identified an inherent challenge obstructing the fabrication of high‐quality Cs‐FA perovskite films using 2ME‐based ink with a mixture of raw powders (PbI_2_, FAI, and CsI), even when using these raw materials of highest available purities (99.99% or greater). The formation of colloidal particles in the 2ME‐based Cs_0.05_FA_0.95_PbI_3_ ink is found to be the key factor responsible for inferior film quality. We unraveled these colloidal particles acting as heterogeneous nucleation centers, continuously generating new crystals, thereby competing with the growth of the pre‐existing crystals. This deficiency of crystal growth is identified to create undesirable microstructural properties within the resulting film, including reduced grain/crystal size, diminished film crystallinity, and the formation of local defective clusters. Such an issue is resolved through the utilization of pre‐synthesized Cs_0.05_FA_0.95_PbI_3_ single crystals as precursors dissolved in the solvents. This approach forms a clean ink free of large (with size > 5 nm) colloidal particles, which leads to suppressed secondary nucleation and extended growth of crystals, resulting ultimately in improved film crystallinity and improvement in film optoelectronic qualities. These improvements transfer to the fabrication of fully printed perovskite photovoltaics with carbon electrodes with a PCE of 19.3% achieved for solar cells and 16.2% for mini solar modules, both standing at the cutting‐edge levels among the fully printed counterparts. The employment of low‐grade raw materials in the facile synthesis of single crystals combined with the improvement of solar cell PCE and stability highlights the cost‐effectiveness of our novel single‐crystal ink strategy as a potential future reliable fabrication method.

## 4 Experimental Section

### Synthesis of Single Crystals

For single crystal synthesis, low‐purity PbI_2_ (99%, Sigma–Aldrich) and CsI (99.9%, Sigma–Aldrich) were used. To prepare the precursor solution for single crystal growth, 2.213 g PbI_2_, 0.784 g FAI, and 0.062 g CsI were co‐dissolved in 4 mL GBL to obtain a 1.2 m solution. 2%_vol_ of formic acid (FAH) was added to the solution, followed by agitation for 10 min. The solution was continuously stirred overnight to ensure full dissolution of the raw powders and was then filtered with a 0.45 µm PTFE filter into a clear 20 mL vial. The filtered solution was transferred to several clean, smaller vials that had flat bottoms. These solutions were moved to a crystal growth setup with precise control of temperature. The temperature was first adjusted to 80 °C and maintained for 1 h, followed by a slow increase to 130 °C at a rate of 2 °C h^−1^. During the temperature increase procedure, a few faceted crystals with a typical size of up to 6 mm were formed from the precursor solution. After keeping to solution at 130 °C for 1 h, the crystals were taken out of the solution, quickly dried on paper wipes, and were completely dried in a vacuum chamber.

### Ink Formation and Characterization

To prepare the 2ME‐based, powder‐mix ink of Cs_0.05_FA_0.95_PbI_3_, 16.9 mg of CsI (99.999%, Sigma–Aldrich), 599.3 mg of PbI_2_ (99.999%, Sigma–Aldrich) and 212.4 mg of FAI were co‐dissolved in a mixture of 0.9 mL 2ME (99.8%, Sigma–Aldrich) and 0.1 mL NMP (99.8%, Sigma–Aldrich) to form a 1.3 m (mol L^−1^) solution. For single‐crystal ink, the Cs_0.05_FA_0.95_PbI_3_ single crystals were dissolved in the corresponding amount of 2ME/NMP mixed solution (9:1 in volume ratio) to form a 1.3 m solution. Both solutions were ultrasonicated for 20 min to ensure that the precursors were fully dissolved. To prepare DMF‐based ink, the same quantities of precursors were alternatively dissolved in a mixture of DMF (99.8%, Sigma–Aldrich)/NMP (9:1 in volume ratio), followed by the same procedure of ultrasonication treatment.

For the powder‐mix ink of Cs_0_FAPbI_3_, Cs_0.02_FA_0.98_PbI_3_, and Cs_0.1_FA_0.9_PbI_3_, the preparation procedure was the same except that the quantities of raw powders were adjusted according to CsI/FAI/PbI_2_ = 0/223.6/599.3 mg, 6.8/219.1/599.3 mg and 33.8/201.2/599.3 mg, respectively. For MA_0.1_FA_0.9_PbI_3_ and MA_0.25_FA_0.75_PbI_3_, the raw powders were co‐dissolved in 1 mL of 2ME/NMP (9:1 volume ratio) according to MAI (99.99%, great cell solar)/FAI/PbI_2_ = 20.7/201.2/599.3 mg and 51.7/167.7/599.3 mg.

Absorption spectra of the precursor inks were measured with a UV–VIS–NIR spectrometer (Lambda 950, Perkin Elmer). The concentration of the sample ink was 0.145 m, diluted from the original ink using either 2ME or DMF and measured with a quartz cuvette. Colloidal sizes of the precursor inks were measured with a dynamic light scattering particle size analyzer (NANO ZS). The concentration of the sample ink was 0.145 m, diluted directly from the original ink without infiltration, and was let stand for 1 min before starting the measurement.

### Thin‐Film Perovskite Fabrication

Perovskite thin films were fabricated by blade coating 15 µL of precursor ink on 2.5  ×  2.5 cm substrate at room temperature (≤ 22 °C). The distance between the coating bar and substrate was 150 µm and the coating speed was 3 mm s^−1^. The liquid film was then dried with continuous airflow for 30 s until the film turned brown. The airflow was provided by a compressed air gun with a pressure of 1.25 bar, and the air gun was ≈20 cm above the substrate with the nozzle perpendicular to the substrate. For 2ME‐based ink, the coating temperature was controlled < 25 °C, and the wet film was treated by air flow immediately after wet‐film casting. Subsequently, the gas‐quenched films were annealed at 100 °C for 10 min and 150 °C for 5 min before coating HTL. The deposition of perovskite films was carried in a dry box with controlled humidity (< 10% RH).

### Solar Cell Fabrication and Measurement

The indium‐doped tin oxide (ITO)‐coated glass substrates were sequentially cleaned by acetone, isopropanol, and deionized water in an ultrasonic bath for 15 min, respectively, and were dried with airflow. All substrates were treated with UV–ozone for 20 min prior to layer coating. The SnO_2_ solution was prepared by directly diluting an aqueous dispersion of colloidal nanoparticles (15%_wt_, Alfa Aesar) with deionized water to form a 6%_wt_ dispersion. The diluted SnO_2_ solution was ultrasonicated for 20 min and was then infiltrated by a microporous Polytetrafluorethylen (PTFE) filter with a pore size of 0.45 µm. The SnO_2_ layer was deposited by blade‐coating the aqueous dispersion at 80 °C, with a bar height of 150 µm and a coating speed of 8 mm s^−1^. The SnO_2_ films were then annealed at 150 °C for 30 min. Both deposition and annealing were carried out in ambient conditions (20–25 °C, 30–40% RH).

TaTm solution was prepared by dissolving 5 mg of TaTm powder (Dyenamo AB) in 1 mL of chlorobenzene (99.8%, Sigma–Aldrich). Prior to deposition, the solution needed to be stirred at 70 °C on a hot plate for at least 20 min to ensure full dissolution of the powder. The solution was then blade‐coated onto perovskite film at 65 °C with a bar height of 150 µm, and a coating speed of 5 mm s^−1^. The toluene‐dispersed PEDOT (Heraeus Clevios HTL Solar 3) was used as received. The dispersion was blade‐coated at 40 °C with a bar height of 150 µm and a coating speed of 10 mm s^−1^. The bilayer HTL was then annealed at 100 °C for 5 m.

Carbon electrodes were printed from a commercial paste (Dyenamo Ltd.) on the HTL. The pixel shape of defined with a laser‐patterned tape mask. The pixel area was defined to be 0.06 cm^2^ and was checked by an optical microscope. Wet carbon paste was subsequently annealed on a hot plate at 120 °C for 15 min in ambient air to fully solidify the carbon electrode.

J–V measurement of the solar cells was carried out with a source measurement system. The 1‐Sun equivalent illumination was provided by an AAA class solar simulator with an AM 1.5G filter (LOT‐Quantum Design). The intensity was calibrated with a commercial reference silicon photodiode certified by PV Calibration Facility Nijmegen. The scan rate is fixed at 100 mV s^−1^ for both reverse scan (1.2 to −0.1 V) and forward scan (−0.1 to 1.2 V). The MPP was determined by a quasi‐steady‐state JV scan at 1 mV s^−1^ in the range near the open circuit. The stabilized PCE was measured by holding the solar cell at the voltage of MPP and recording the photocurrent for 300 s. All solar cells were measured in a nitrogen‐filled chamber with a glass window at room temperature.

### Solar Cell Stability Measurement

The aging of the solar cells was carried out in a nitrogen‐filled chamber with a glass window. The chamber was kept at 85 ±  5 °C assisted by an underneath hot plate and was calibrated with a thermal couple. The continuous illumination was provided with a metal halide lamp and a UV filter was placed in front of the solar cells. The light intensity was ≈1 Sun equivalent and was continuously monitored by a photodiode sensor. Both solar cells were held at a fixed voltage that is close to the voltage of their MPPs. JV scans (in both forward and reverse directions) were carried out periodically (every ≈20 min) to determine the PCE of the solar cells.

### In Situ PL Characterization

PL measurement of the liquid films during the drying process was carried out with a home‐built confocal spectroscopic setup. A 532 nm laser diode was used as an excitation source, and a 550 nm long‐pass filter was placed before the spectrometer. The measurement was turned on simultaneously with the gas quenching treatment, and signals were acquired every 0.5 s till the end of the gas quenching treatment.

### Optical Microscope, Confocal Microscope, XRD and SEM

Optical microscopic images of the perovskite film were taken with an MX51 microscope (Olympus Co.), using light‐transmitting mode and a 100 × magnification lens. Macroscopic surface roughness of the perovskite films was measured with a Nanofocus confocal microscope set up, using a 100 × magnification lens under the µsurf 3Dd topometry mode. The surface morphology of perovskite films was characterized by a JEOL JSM7610F scanning electron microscopy. XRD patterns of perovskite films were measured with a Panalytical X'pert powder diffractometer with filtered Cu‐Kα and an X'Celerator solid‐state stripe detector operated at 40 kV and 30 mA. The thin‐film sample was kept rotatory during the measurement. The XRD pattern of the single crystal top facet was measured the same way.

### Pc‐AFM Measurements

Conducting probe AFM measurements were performed using a Bruker Dimension icon with ScanAsyst (Nanoscope‐6 controller). Prior to the measurements, the current amplifier was calibrated using a 10 MΩ resistor in a wide range of current sensitivities (1pA to 100 nA). Current mapping was performed using platinum and iridium‐coated tips (SCM‐PIT‐V2, Bruker) with a force constant of ≈ 3 N m^−1^ having 250 kHz resonance frequency. During the measurements, −2 V bias was applied to the sample. Photocurrent mapping was carried out under bottom illumination using a Redoxme LED light source (λ = 529 nm) connected to the multimode optical fiber. The intensity of the fiber output was determined by the PM100A power meter (Thorlabs) connected to the S120VC (Thorlabs) optical sensor. 

### Solar Module Fabrication and Measurement

Perovskite mini solar modules were fabricated on 5  ×  5 cm^2^ ITO‐coated glass substrate. P1 lines were patterned by femtosecond laser scribing. The patterned substrates were then cleaned and treated with UV Ozone and were deposited with SnO_2_, perovskite, TaTm, and PEDOT sequentially. All these processes were the same as solar cell fabrication, except that the volume of solution used for blade coating is double of the volume for solar cell fabrication. Following the layer deposition, P2 lines were patterned with femtosecond laser scribing. The quality of P2 lines was examined with an optical microscope to ensure SnO_2_ was fully removed whilst the ITO layer remained unaffected. Carbon electrode was printed with the same method as solar cells. The P3 line was fabricated by attaching a pre‐patterned tape mask on the surface of the mini solar module prior to the coating carbon paste. JV characteristics of the mini solar modules were carried out with the same solar simulator but in ambient conditions. The measurement protocol of reverse and forward JV scan, MPP determination, and stabilized PCE measurement were the same as measuring solar cells. The solar modules were tested in ambient conditions without encapsulation.

## Conflict of Interest

The authors declare no conflict of interest.

## Author Contributions

T.D., C.J.B., and H.J.E conceived the idea and designed the experiments. T.D. carried out the optimization of perovskite film printing, solar cell, and solar module fabrication/characterization, and in situ photoluminescence measurements. V.R. optimized single crystal synthesis, single crystal characterization, and solution characterization that is supervised by W.H. S.Q. assisted and optimized solar module fabrication. S.P. and H.Y. carried out pc‐AFM measurement and were supervised by J.B. D.J. assisted in situ measurement setup. Z.P. and J.Z. set up and conducted stability measurements of the devices. All authors discussed the results and commented on the manuscript.

## Supporting information

Supporting Information

## Data Availability

The data that support the findings of this study are available from the corresponding author upon reasonable request.
