# Supplementary material for: Precursor‐Engineered Volatile Inks Enable Reliable Blade‐Coating of Cesium–Formamidinium Perovskites Toward Fully Printed Solar Modules
Source: Adv Sci (Weinh). 2024 May 13;11(28):2401783. doi: 10.1002/advs.202401783 (PMC11267368; doi:10.1002/advs.202401783)
Supplement: Supplementary file 1 — Supporting Information [file ADVS-11-2401783-s001.pdf]

ADVANCED SCIENCE
Open Access

Supporting Information

for *Adv. Sci.*, DOI 10.1002/advs.202401783

Precursor-Engineered Volatile Inks Enable Reliable Blade-Coating of Cesium–Formamidinium Perovskites Toward Fully Printed Solar Modules

Tian Du, Viktor Rehm, Shudi Qiu, Subhajit Pal, Dongju Jang, Zijian Peng, Jiyun Zhang, Haozhen Yuan, Joe Briscoe, Wolfgang Heiss, Christoph J. Brabec* and Hans-Joachim Egelhaaf*

Precursor-Engineered Volatile Inks Enable Reliable Blade-Coating of Caesium-Formamidinium Perovskites Towards Fully Printed Solar Modules

Tian Du[1,2,*], Viktor Rehm[2], Shudi Qiu[2], Subhajit Pal[3], Dongju Jang[2], Zijian Peng[1,2], Jiyun Zhang[1,2], Haozhen Yuan[3], Joe Briscoe[3], Wolfgang Heiss[2], Christoph J. Brabec[1,2,*], Hans-Joachim Egelhaaf[1,2]

[1]Forschungszentrum Jülich GmbH, Helmholtz-Institute Erlangen-Nürnberg (HI ERN), Immerwahrstraße 2, 91058 Erlangen, Germany

[2]Department of Material Science, Materials for Electronics and Energy Technology (i-MEET), Friedrich-Alexander-Universität Erlangen-Nürnberg, Martensstraße 7, 91058 Erlangen, Germany

[3]School of Engineering and Materials Science, Queen Mary University of London, London E1 4NS, United Kingdom

Supplementary figures

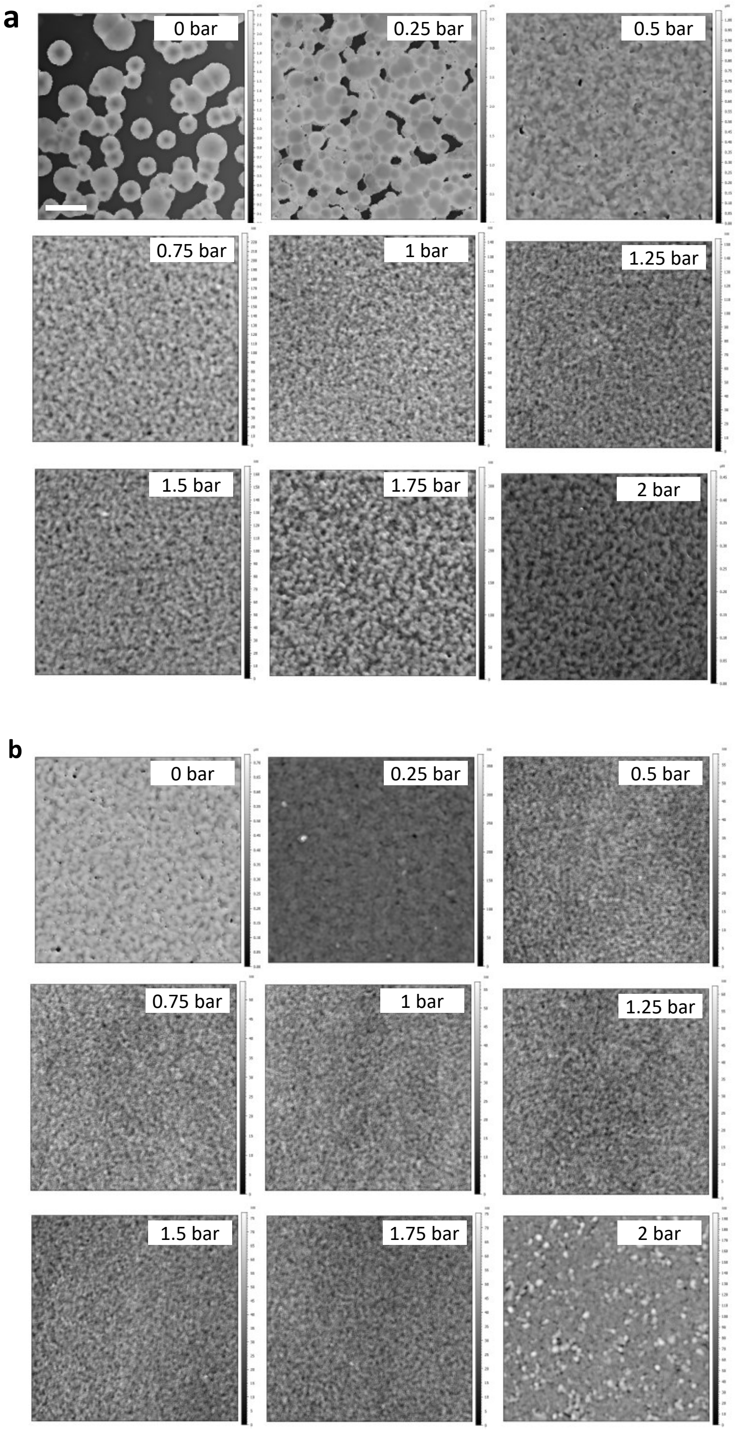

Figure S1. Confocal microscopic images showing the surface height variation of perovskite films prepared from (a) DMF-based inks and (b) 2ME-based inks with gas-quenching treatment of varied gas pressure. Surface roughness is calculated automatically from these images. The scale bar is 400 nm for all figures.

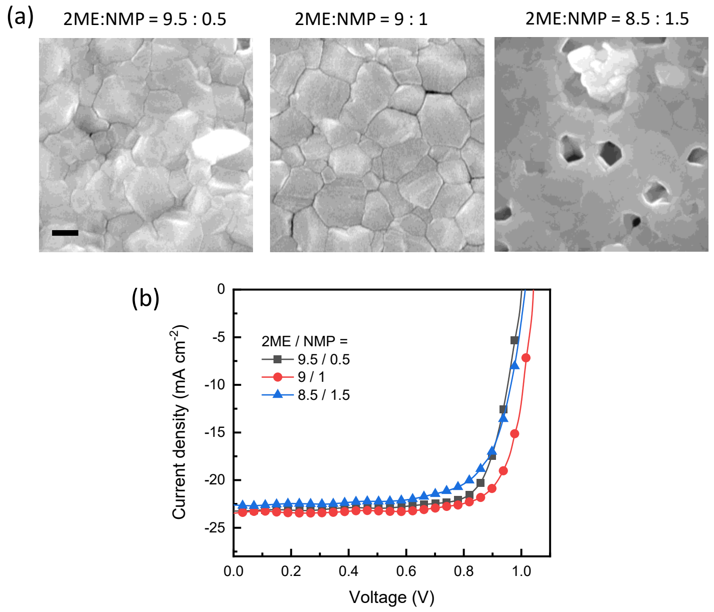

Figure S2. (a) Surface SEM images of perovskite films processed from single-crystal inks with varied 2ME : NMP volume ratios of 9.5 : 0.5, 9 : 1 and 8.5 : 0.5. The scale bar is 100 nm. (b) Representative JV curves of solar cells comprising these perovskite films.

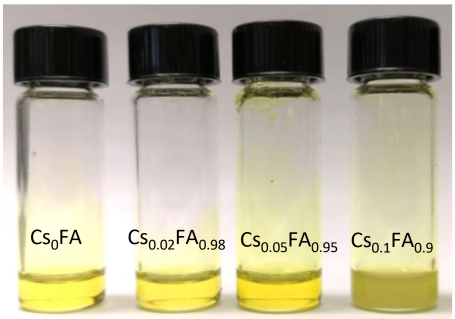

Figure S3. Photograph of 2ME-based inks, after ultrasonicating for 30 minutes, with mixed raw powders of different composition: $FAPbI_3$, $Cs_{0.02}FA_{0.98}PbI_3$, $Cs_{0.05}FA_{0.95}PbI_3$ and $Cs_{0.1}FA_{0.9}PbI_3$.

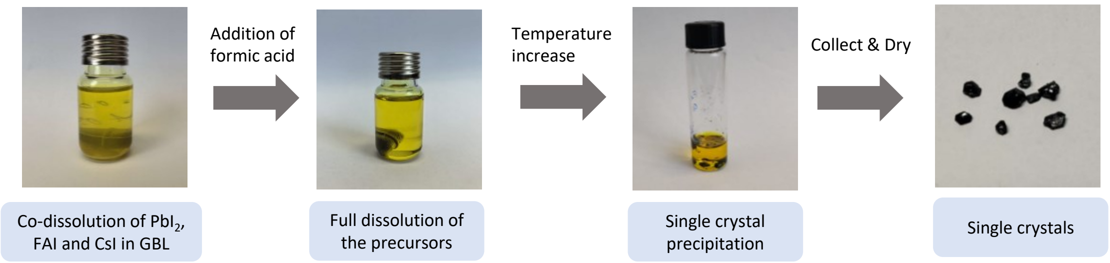

Figure S4. Photographs at different stages of single crystal synthesis.

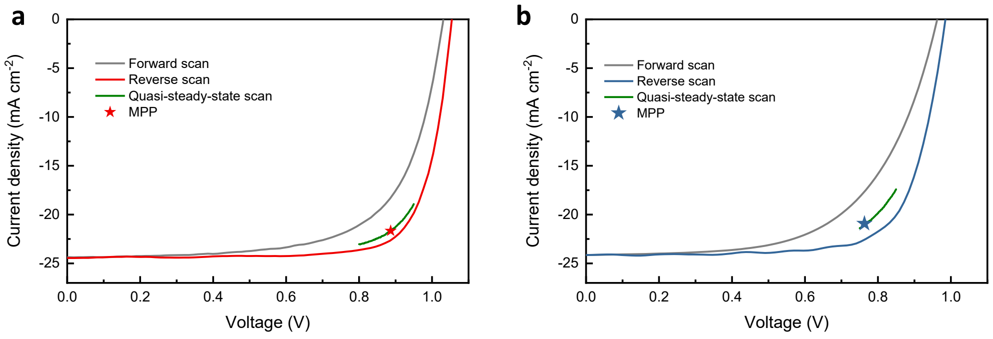

Figure S5. JV curves measured from "reverse scan" (open circuit to short circuit, 100 mV s^{-1}), "forward scan" (short circuit to open circuit, 100 mV s^{-1}), quasi-steady-state scan (1 to 0.8 V or 0.85 to 0.75 V, 1 mV s^{-1}) for the champion devices with perovskite films derived from single-crystal ink (a) and powder-mix ink (b). The maximum power points (MPP), indicated by star symbols, are determined from the quasi-steady-state scan for both devices.

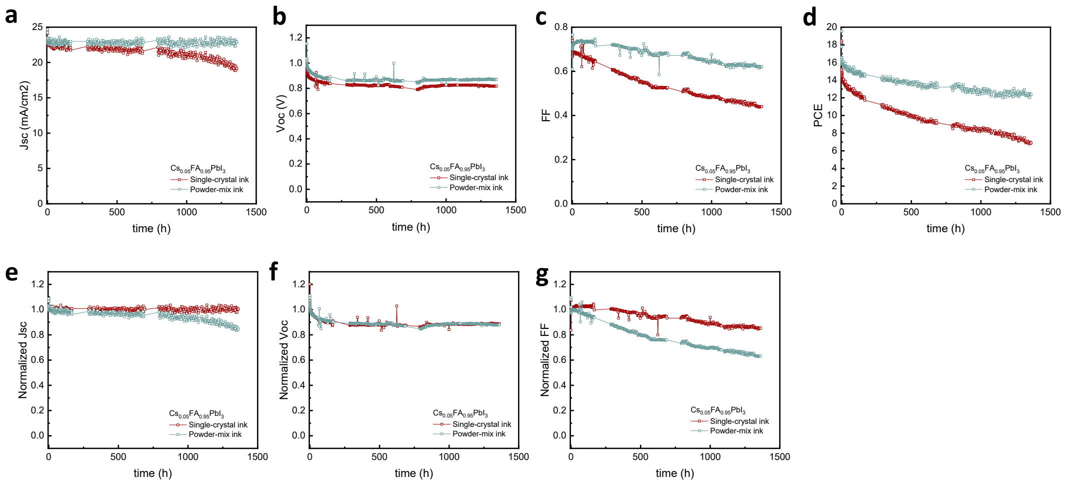

Figure S6. Evolution of PV parameters during ageing test: **(a)** J_{SC}, **(b)** V_{OC}, **(c)** FF and **(d)** PCE. **(e – g)** Normalized values of J_{SC}, V_{OC} and FF.

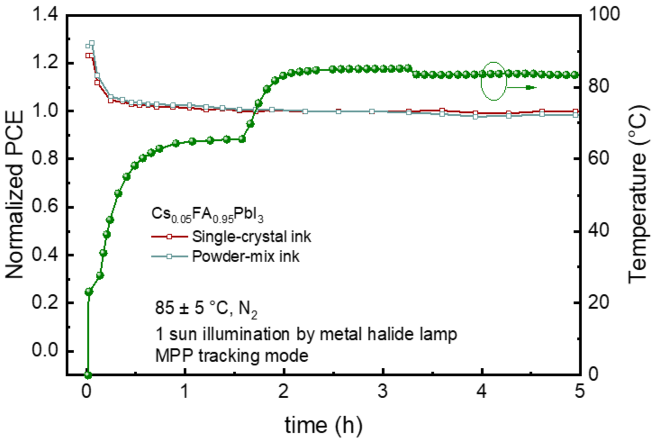

Figure S7. Evolution of normalized PCE and the temperature of the measurement chamber in the initial 5 hours of ageing test.

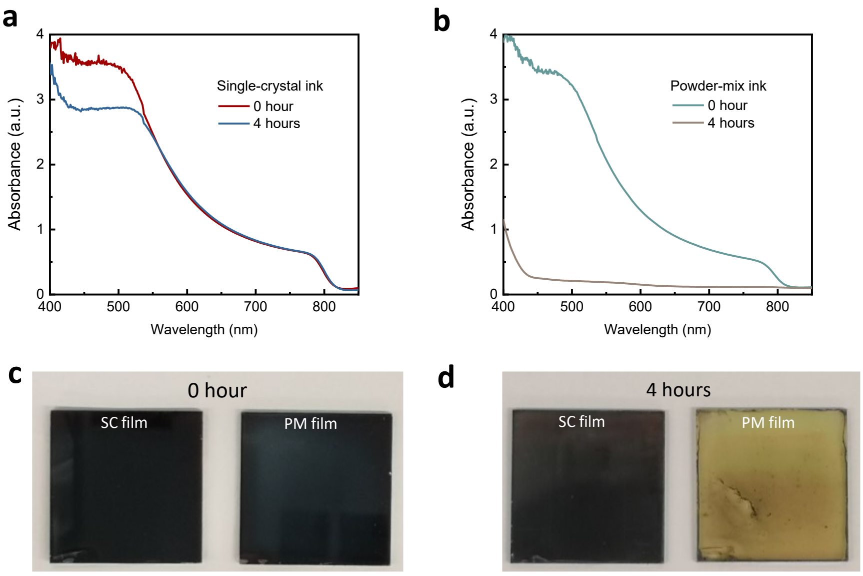

Figure S8. (a – b) Absorption spectra of $Cs_{0.05}FA_{0.95}PbI_3$ perovskite films measured before (0 hour) and after ageing in ambient condition for 4 hours (40 – 60 %RH, with indoor room light). **(c – d)** Photographs of perovskite films before and after ageing.

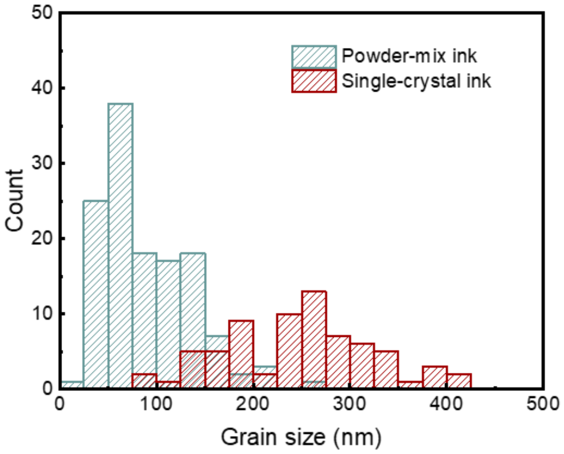

Figure S9. Statistical data of grain size measured from the SEM images.

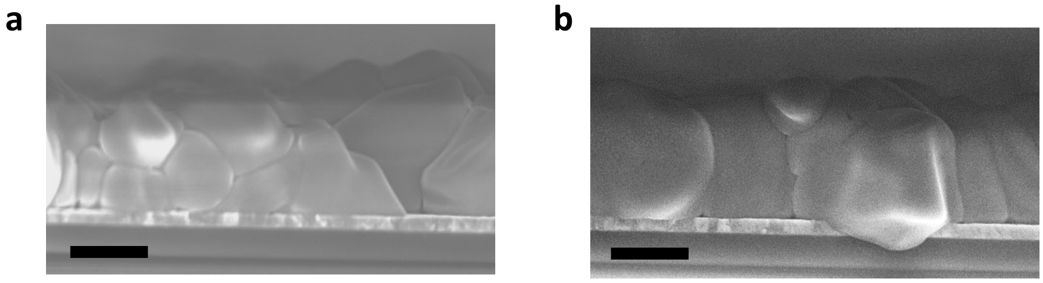

Figure S10. Cross-sectional SEM images of the perovskite films processed from (a) powder-mix ink and (b) single-crystal ink. The scale bars are 500 nm in both images.

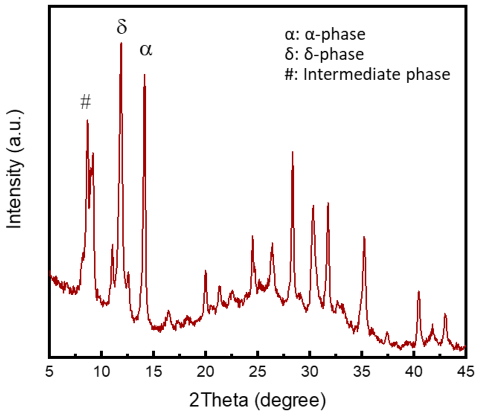

Figure 11. XRD pattern of CsFA perovskite film after gas quenching before thermal annealing. The characteristic peaks of α-phase, δ-phase and the so-called intermediate phase are marked on the pattern.

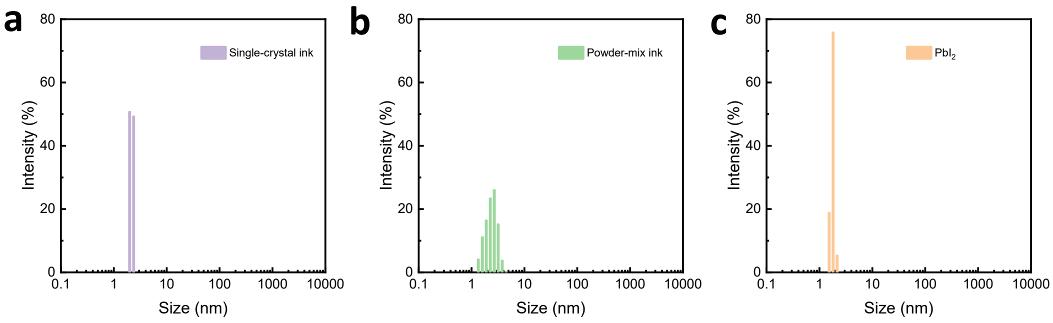

Figure S12. Colloidal hydrodynamic size distribution of DMF-based perovskite inks with **(a)** single crystals $Cs_{0.05}FA_{0.95}PbI_3$, **(b)** powder mix $Cs_{0.05}FA_{0.95}PbI_3$ and **(c)** PbI_2 only.

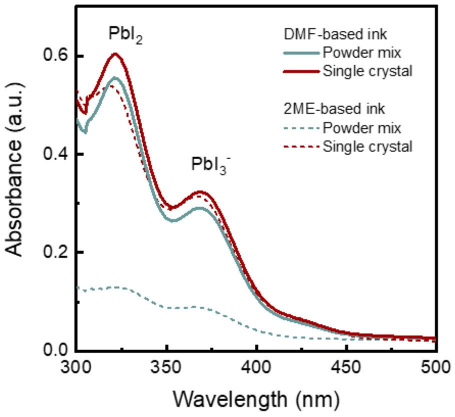

Figure S13. Absorption spectra of DMF-based perovskite inks with single crystals and powder mix.

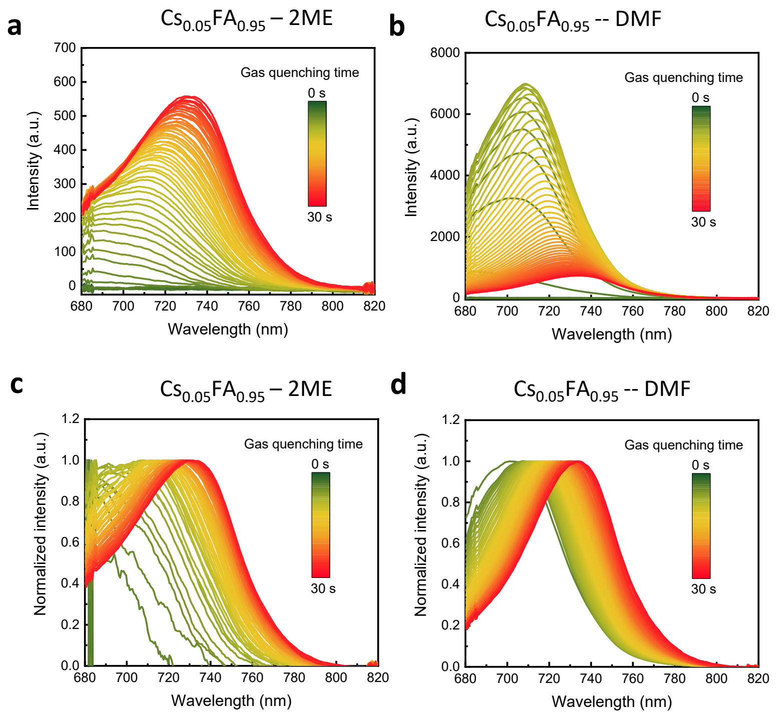

Figure S14. Comparison of spectral evolution of PL of the wet films cast from a **(a)** 2ME-based ink and **(b)** a DMF-based ink, and their normalized spectra **(c – d)**, both with mixed powder mix as precursors.

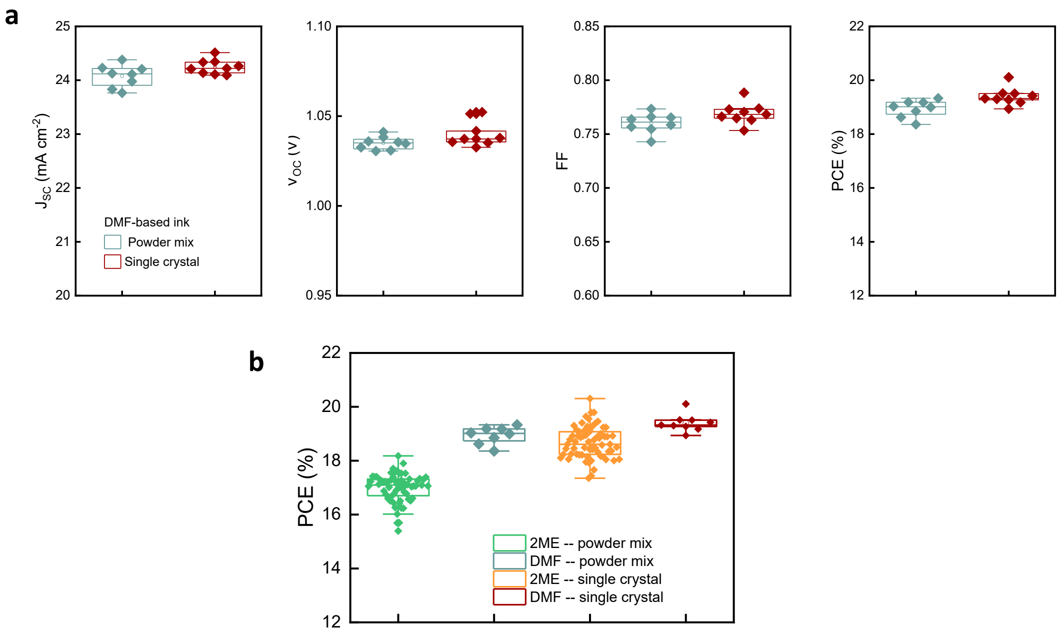

Figure S15. (a) Statistical data of solar cell performance fabricated with DMF-based inks containing mixed raw powders or single crystals as precursors. **(b)** Comparison of solar cell PCE between perovskite films processed from 2ME-based ink and DMF-based ink using both powder mix and single crystal as precursors.

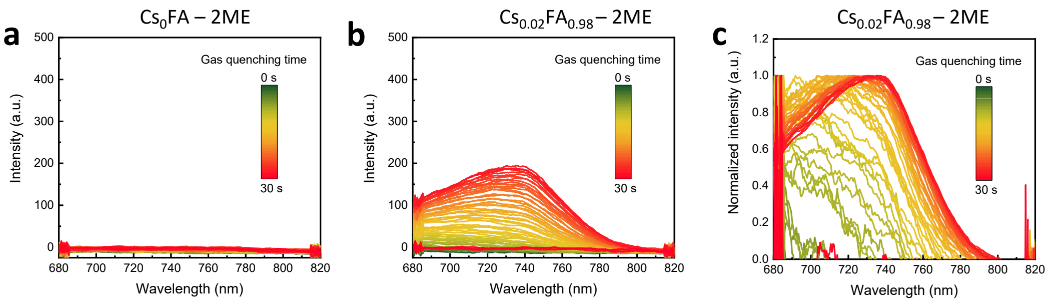

Figure S16. Spectral evolution of PL of **(a)** Cs_0FA and **(b)** $Cs_{0.02}FA_{0.98}$ wet films and **(c)** normalized spectra for $Cs_{0.02}FA_{0.98}$, cast from 2ME-based inks, during gas quenching treatment.

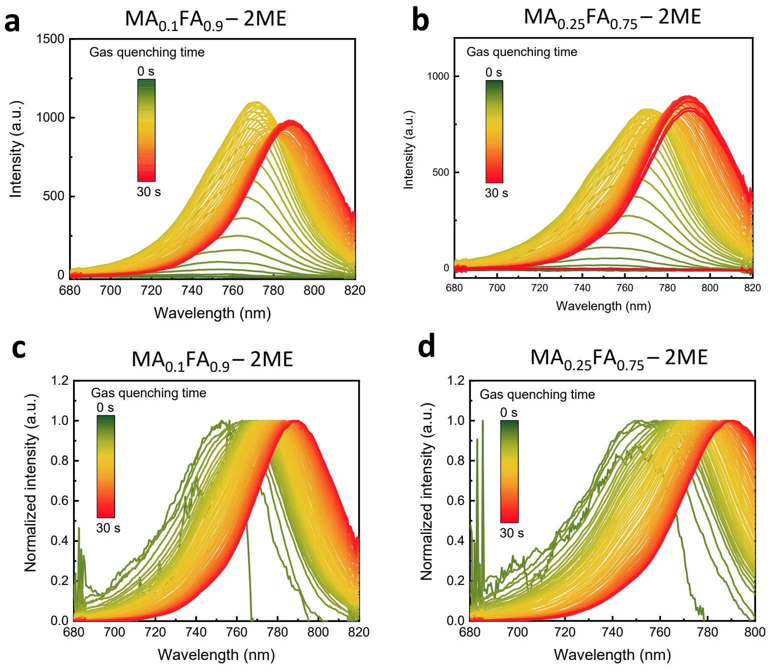

Figure S17. Spectral evolution of PL of **(a)** $MA_{0.1}FA_{0.9}$ and **(c)** $MA_{0.25}FA_{0.75}$ wet films and their normalized spectra **(c – d)**, cast from 2ME-based inks, during gas quenching treatment.

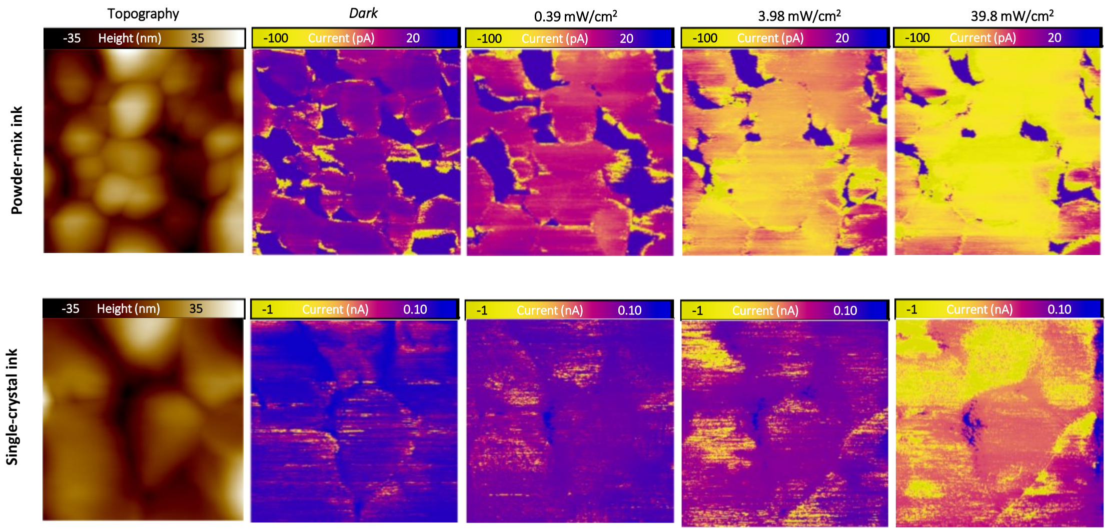

Figure S18. Topographic maps and current maps measured under dark and varied light intensities. The bias applied on tip is – 1.5 V.

Table S1. Photovoltaic parameters and stabilized PCE of 12 perovskite mini solar modules.

Number	I_{sc} / mA	V_{OC} / V	FF	PCE / % (measured from JV scan)	Stabilized PCE / %
1	56.7	7.153	0.71	16.95	15.71
2	56.9	7.044	0.73	17.38	16.11
3	57.4	7.013	0.72	17.13	15.78
4	56.0	6.937	0.71	16.34	15.62
5	56.5	7.055	0.71	16.85	15.55
6	56.1	7.027	0.74	17.21	15.83
7	56.6	7.021	0.71	16.69	15.10
8	56.7	7.005	0.73	17.07	15.52
9	56.8	7.067	0.74	17.69	16.20
10	55.1	7.043	0.72	16.66	15.35
11	55.6	6.808	0.72	16.21	15.14
12	54.7	7.014	0.71	16.09	14.75

Table S2. Summary of active area, configuration, processing temperature, PCE and year of publication of fully printed perovskite solar modules reported in literature.

Reference	Year	Active area / cm^2	Configuration	Maximum Processing temperature / °C	PCE / %
**This work**	**--**	**16.84**	**ITO/SnO_2/$Cs_{0.05}FA_{0.95}PbI_3$/TaTm/PEDOT/Carbon**	**150**	**17.69 (stabilized 16.20)**
[1]	2024	16.84	ITO/SnO_2/$MAPbI_3$ /PEDOT/Carbon	120	15.28
[2]	2021	7.2	ITO/SnO_2/$GA_xMA_{1-x}PbI_3$/PDCBT/P3HT/Carbon	120	15.3
[3]	2019	22.4	FTO/c-TiO_2/$MAPbI_3$/Carbon doped with CuPC	100	7.2
[4]	2017	17.6	FTO/ZnO/$MAPbI_3$/Carbon	120	10.6
[5]	2021	4.32	FTO/c-TiO_2/m-TiO_2/m-ZrO_2/m-Carbon/ (5-AVA)$_x$$MA_{1-x}PbI_3$	400	8.7
[6]	2021	70	FTO/c-TiO_2/m-TiO_2/m-ZrO_2/m-Carbon/ (5-AVA)$_x$$MA_{1-x}PbI_3$	395	11.55
[7]	2020	60.08	FTO/c-TiO_2/m-TiO_2/m-ZrO_2/m-Carbon/ (5-AVA)$_x$$MA_{1-x}PbI_3$	400	12.87
[8]	2017	49	FTO/c-TiO_2/m-TiO_2/m-ZrO_2/m-Carbon/ (5-AVA)$_x$$MA_{1-x}PbI_3$	400	10.4
[9]	2016	70	FTO/c-TiO_2/m-TiO_2/m-ZrO_2/m-Carbon/ (5-AVA)$_x$$MA_{1-x}PbI_3$	400	10.74
[10]	2016	47.6	FTO/c-TiO_2/m-TiO_2/m-ZrO_2/m-Carbon/ (5-AVA)$_x$$MA_{1-x}PbI_3$	400	11.16

Table S3. Manufacturer, purity and catalogue price of the raw materials for single crystal synthesis and powder-mix precursor preparation.

	Material	Manufacturer	Purity / %	Catalogue Price	Quantity / g	Catalogue price per gram / euro
**Powder mix**	CsI	Merck	99.999	136	10	13.60
	PbI_2	Merck	99.999	157	5	31.40
	FAI	Greatcell Solar	99.990	6033	5000	1.21
**Single crystal**	CsI	Merck	99.9	276	100	2.76
	PbI_2	Merck	99	86	50	1.72

GBL	Merck	99	613	20000	0.03
Formic Acid	Themo Fisher Scientific	97	153	10000	0.02
FAI	Greatcell Solar	99.990	6033	5000	1.21

Table S4. Cost of the precursor material for $Cs_{0.05}FA_{0.95}PbI_3$ single crystal and $Cs_{0.05}FA_{0.95}PbI_3$ powder mix.

	Material	Cost per gram of $Cs_{0.05}FA_{0.95}PbI_3$ / euro	Total price per gram of $Cs_{0.05}FA_{0.95}PbI_3$ / euro
	CsI	0.2856	
**Powder mix**	PbI_2	22.7022	**23.30**
	FAI	0.30889	
	CsI	0.1518	
	PbI_2	3.37042	
**Single crystal (Yield = 37%)**	GBL	0.1226	**4.48**
	Formic Acid	0.00133	
	FAI	0.834	

Reference

[1] S. Qiu, M. Majewski, L. Dong, D. Jang, V. M. L. Corre, J. G. Cerrillo, O. J. J. Ronsin, F. Yang, F. Guo, K. Zhang, L. Lüer, J. Harting, T. Du, C. J. Brabec, H. J. Egelhaaf, *Adv. Energy Mater.* **2024**, *14*, 2303210.

[2] F. Yang, L. Dong, D. Jang, B. Saparov, K. C. Tam, K. Zhang, N. Li, C. J. Brabec, H. J. Egelhaaf, *Adv. Energy Mater.* **2021**, *11*, 2101219.

[3] S. He, L. Qiu, D. Y. Son, Z. Liu, E. J. Juarez-Perez, L. K. Ono, C. Stecker, Y. Qi, *ACS Energy Lett.* **2019**, *4*, 2032–2039.

[4] L. Cai, L. Liang, J. Wu, B. Ding, L. Gao, B. Fan, *J. Semicond.* **2017**, *38*, 014006.

[5] E. Kobayashi, R. Tsuji, D. Martineau, A. Hinsch, S. Ito, *Cell Reports Phys. Sci.* **2021**, *2*, 100648.

[6] K. S. Keremane, S. Prathapani, L. J. Haur, A. Bruno, A. Priyadarshi, A. V. Adhikari, S. G. Mhaisalkar, *ACS Appl. Energy Mater.* **2021**, *4*, 249–258.

[7] M. Xu, W. Ji, Y. Sheng, Y. Wu, H. Cheng, J. Meng, Z. Yan, J. Xu, A. Mei, Y. Hu, Y. Rong, H. Han,

Nano Energy **2020**, *74*, 104842.

[8] Y. Hu, S. Si, A. Mei, Y. Rong, H. Liu, X. Li, H. Han, *Sol. RRL* **2017**, *1*, 1600019.

[9] A. Priyadarshi, L. J. Haur, P. Murray, D. Fu, S. Kulkarni, G. Xing, T. C. Sum, N. Mathews, S. G. Mhaisalkar, *Energy Environ. Sci.* **2016**, *9*, 3687–3692.

[10] G. Grancini, C. Roldán-Carmona, I. Zimmermann, E. Mosconi, X. Lee, D. Martineau, S. Narbey, F. Oswald, F. De Angelis, M. Graetzel, M. K. Nazeeruddin, *Nat. Commun.* **2017**, *8*, 15684.
